# Characterization of Surface Receptor Expression and Cytotoxicity of Human NK Cells and NK Cell Subsets in Overweight and Obese Humans

**DOI:** 10.3389/fimmu.2020.573200

**Published:** 2020-09-23

**Authors:** Wiebke Naujoks, Dagmar Quandt, Anja Hauffe, Heike Kielstein, Ina Bähr, Julia Spielmann

**Affiliations:** ^1^Institute of Anatomy and Cell Biology, Medical Faculty of Martin Luther University Halle-Wittenberg, Halle (Saale), Germany; ^2^Regenerative Medicine Institute (REMEDI) at CÚRAM Centre for Research in Medical Devices, School of Medicine, College of Medicine, Nursing and Health Sciences, National University of Ireland Galway, Galway, Ireland

**Keywords:** NK cells, overweight, obesity, cytotoxicity, NK cell receptors, NK cell subsets, breast

## Abstract

Obesity is associated with an increased risk for several cancer types and an altered phenotype and functionality of natural killer (NK) cells. This study aimed to investigate the association of overweight and obesity with NK cell functions and receptor expression profiles in humans. Therefore, peripheral blood mononuclear cells were isolated from normal weight, overweight, and obese healthy blood donors. In depth analysis of immune cell populations and 23 different surface markers, including NK cell receptors, NK-cell-related markers as well as functional intracellular markers on total NK cells and NK subgroups were performed by multicolor flow cytometry. The data revealed a decreased expression of the activating NK cell receptors KIR2DS4 and NKp46 as well as an increased expression of the inhibitory NK cell receptors NKG2A and Siglec-7 in overweight and obese compared to normal weight individuals. Additionally, the expression of the adhesion molecule CD62L and the maturation and differentiation marker CD27 was downregulated in NK cells of overweight and obese subjects. Furthermore, the cytotoxicity of NK cells against colorectal cancer cells was decreased in overweight and obese subjects. Investigations on underlying killing mechanisms demonstrated a reduced TRAIL expression on NK cells of obese subjects suggesting an impaired death receptor pathway in obesity. The present study gives new insights into an impaired functionality and phenotype of NK cells and NK cell subsets in overweight and obesity. These phenotypic alterations and dysfunction of NK cells might be an explanation for the increased cancer risk in obesity.

## Introduction

Overweight and obesity have been defined by the World Health Organization (WHO) as “abnormal or excessive fat accumulation that presents a risk to health” (1). For adults, the most commonly used and practical method to measure overweight and obesity is the body mass index (BMI). The BMI is defined as the weight in kilograms divided by the square of the height in meters (kg/m^2^). According to the WHO definitions, a BMI of 18.5–24.9 kg/m^2^ is defined as normal weight, a BMI of 25–29.9 kg/m^2^ is considered overweight, and a BMI of ≥30 kg/m^2^ is defined as obese. The prevalence of obesity is continuously rising, reaching pandemic levels with worldwide 39% of adults being overweight and 13% being obese in 2016 (1). Besides the higher risk for several non-communicable diseases, obesity is associated with an increased susceptibility to infections and a higher risk for several cancer types including the most common cancers among men and women – colorectal cancer (CRC) and postmenopausal breast cancer (2–5). Although a number of studies have demonstrated that overweight and early obese states can improve cancer survival, numerous studies show that severe obesity is associated with an increased cancer mortality (6–8). The underlying biological mechanisms that link obesity and cancer still remain unclear. Besides genetic factors, unhealthy diet, reduced physical inactivity, obesity-related insulin resistance, increased secretion of sex hormones and cytokines by adipose tissue, also the chronic low-grade inflammation and obesity-associated alterations of immune cell function are discussed as possible mechanisms underlying the increased risk for several cancer types in obesity (9–14).

Excess fat accumulation results in a dysregulated secretion of adipokines, i.e., leptin and adiponectin, as well as an infiltration of adipose tissue by inflammatory immune cells accompanied with an increased secretion of proinflammatory cytokines, i.e., interferon (IFN)-γ, interleukin (IL)-6, and tumor necrosis factor (TNF)-α (15). Moreover, obesity is associated with an altered gut microbiota, which has also been shown to be involved in local and systemic inflammatory processes (16, 17). Therefore, obesity is associated with a local and systemic chronic low-grade inflammatory state (18). This so-termed metaflammation leads to dysfunctions of several immune cells, like macrophages, T lymphocytes, and B lymphocytes (19–21). In addition, numerous studies demonstrated an impaired functionality of natural killer (NK) cells in obese animals and humans (22).

Natural killer cells are large granular effector lymphocytes and can rapidly kill virus-infected and malignant transformed cells without prior sensitization while remaining tolerant of normal cells (23). In humans, several NK cell subpopulations can be distinguished, based on the expression density of CD56 and CD16 on their cell surface. The two major and classically defined subsets are the CD56^dim^CD16^bright^ and the CD56^bright^CD16^dim^ NK cell subsets (24, 25). The CD56^dim^CD16^bright^ NK cells subset represents the majority of NK cells in the peripheral blood (∼90%) and is mostly responsible for cytotoxic activity and target cell killing. The CD56^bright^CD16^dim^ NK cell subset represents about 10% of peripheral NK cells and has more immunoregulatory function through their ability to produce abundant cytokines, including IFN-γ, TNF-α and interleukins (24, 26).

Natural killer cells mediate their cytotoxicity against target cells via two direct mechanisms. On the one hand, NK cells secrete their cytotoxic granules, containing pore-forming perforin and apoptosis-inducing granzymes, which induces target cell apoptosis by activating cell-death mechanisms. These include the activation of apoptotic caspases, but also caspase-independent apoptotic pathways. This granule-exocytosis pathway is mediated by the engagement of FcγRIII (CD16) receptor on an antibody-coated target cell or by the engagement of activating receptors on their cognate ligands on the target cell (27–29). On the other hand, direct target cell killing by NK cells is mediated by the stimulation of death receptors, e.g., Fas, on target cells by their corresponding ligands, e.g., Fas ligand (FasL) or TNF-related apoptosis-inducing ligand (TRAIL), expressed on NK cells. This death receptor pathway induces caspase-dependent apoptosis of the target cell (27, 28, 30). Besides their cytotoxic function, activated NK cells secrete a number of cytokines and chemokines in order to co-stimulate other cells of the immune system (31).

NK cells express a variety of receptors and the NK cell effector function is orchestrated by a family of inhibitory and activating surface receptors as well as by adhesion molecules and other functional markers (32). The balance of activating and inhibitory signals dynamically regulate the effector functions of NK cells (23).

In humans, the NK cell receptor repertoire includes three major receptor families: natural cytotoxicity receptors (NCRs) and C-type lectin-like receptors as well as human killer immunoglobulin-like receptors (KIRs). Most relevant activating NK cell receptors comprise the NCRs NKp30, NKp44, and NKp46, the natural killer group (NKG) 2D receptor, DNAM (DNAX accessory molecule)-1 and the short-tail members of KIRs (33–35). Inhibitory receptors expressed on NK cell surface are the long-tail members of the KIR family, NKG2A and the killer cell lectin-like receptor subfamily (KLR) G1, CD161 (also called NKRP1, NK1.1, KLRB1), and Siglec-7 (35–38).

In addition, other surface proteins, such as co-receptors and adhesion molecules, contribute to the regulation of effector functions of NK cells. The surface markers CD2 (LFA-2: lymphocyte function-associated antigen 2) and CD62L (L-selectin) act as adhesion molecules, whereas the homing marker CD62L plays an important role in the migration and infiltration of NK cells to lymph nodes and tissues (39–42). Moreover, known co-stimulatory receptors are 2B4 (CD244) and NKp80, which are co-activating, as well as TIGIT (T-cell immunoreceptor with Ig and ITIM domains) and PD-1 (programmed cell death receptor-1), which are co-inhibitory (43–46). Additional activation-associated markers are CD25, CD69 as well as CD107a (LAMP 1: lysosomal-associated membrane protein 1), which was found to be a marker of degranulation on NK cells (47–49).

To define the maturation and differentiation status of NK cells, the extracellular surface receptors CD16, CD56, CD27, and CD57 as well as the intracellular markers and transcription factors EOMES (eomesodermin), T-bet (T-cell associated transcription factor), and Blimp-1 (B lymphocyte-induced maturation protein-1) are used (50–52).

Recent studies demonstrated a direct influence of adipokines, like leptin and IL-6, on NK cell functionality (53–56). In addition, human and animal studies revealed evidence for obesity-associated alterations of number, phenotype, subset proportions, tissue distribution, cytotoxicity, cytokine secretion, and signaling cascades of NK cells (22). Until now, most of the rare studies investigating the association of obesity with human NK cells have focused mainly on total NK cells. Little research has been done on obesity-related impacts subdividing the two main NK cell subpopulations CD56^bright^CD16^dim^ and CD56^dim^CD16^bright^. In addition, to our knowledge no data exist investigating the association of an overweight (pre-obese) body weight with NK cell receptor expression and NK cell functionality. Furthermore, only a limited number of studies address the link of obesity, NK cells and the increased risk for the most frequent cancers among men and women – CRC and postmenopausal breast cancer, respectively – in overweight and obese individuals. Therefore, the aim of the present study was to analyze the expression of activating and inhibitory surface receptors as well as intracellular and extracellular functional markers on total NK cells and NK cell subsets of normal weight, overweight and obese individuals. Additionally, the BMI-dependent cytolytic activity of primary human NK cells against colon and breast cancer cells was investigated.

## Materials and Methods

### Cell Lines

For functional assays, the human mammary adenocarcinoma cell line MCF-7 (kindly provided by Dr. Matthias Bache, Department of Radiation Oncology, University Hospital Halle (Saale), Halle (Saale), Germany), the human colorectal adenocarcinoma cell line DLD-1 (Sigma-Aldrich, St. Louis, MO, United States), the human NK cell line NK-92 and the leukemia cell line K562 (both kindly provided by Prof. Roland Jacobs, Department of Clinical Immunology and Rheumatology, Hannover Medical School, Hannover, Germany) were used. All cell lines were cultured with RPMI 1640 supplemented with 10% of fetal bovine serum (FBS), 1% penicillin/streptomycin (10,000 U/ml penicillin; 10 μg/ml streptomycin) and 1% sodium-pyruvate (100 mM), further referred to as complete RPMI (cRPMI) and were maintained under standard cell culture conditions. NK-92 cells were additionally supplemented with 200 U/ml recombinant human IL-2 (hIL-2).

### Blood Donors

Leukocyte-enriched buffy coats of 46 blood donors were collected and provided by the Department of Transfusion Medicine at the University Hospital Halle (Saale). Each donor had signed an agreement before blood donation that allowed the use of blood for research purposes and was approved by the ethics committee at the University Hospital in Halle (Saale), Germany. Male blood donors aged over 18 years were included. Exclusion criteria were acute infection, immunosuppression and known malignant tumors. For BMI related analysis blood donors were divided into three groups, based on the given information of body weight and height – normal weight with BMI 18.5–24.9 kg/m^2^, overweight with BMI 25–29.9 kg/m^2^, and obese with BMI ≥30 kg/m^2^.

### Separation of Peripheral Blood Mononuclear Cells From Human Buffy Coats

Isolation of peripheral blood mononuclear cells (PBMCs) from the buffy coats was performed by density gradient centrifugation using Biocoll separation solution (Biochrom, Berlin, Germany) and LeukoSep^TM^ tubes (Greiner Bio-One, Frickenhausen, Germany) followed by an incubation with red blood cell lysis buffer (c.c. pro, Oberdorla, Germany) to remove erythrocytes residues. Purified PBMCs were cryopreserved and stored in liquid nitrogen until further analyzed.

### Multicolor Flow Cytometric Analysis of Human PBMCs

The flow cytometric analysis was used to determine and quantify specific cell populations of human PBMCs, in particular monocytes and lymphocytes, and to characterize the phenotype of NK cells, especially of the two main NK cell subsets CD56^bright^CD16^dim^ and CD56^dim^CD16^bright^ NK cells. Antibodies for surface and intracellular staining of human PBMCs are presented in Supplementary Tables 1, 2.

#### Surface Staining and Determination of Extracellular Markers

Human PBMCs of blood donors from three different BMI groups were stained with an appropriate combination of fluorochrome-conjugated monoclonal antibodies to analyze immune cell populations and NK cell receptor surface expressions by flow cytometry. Several eight-antibody plex panel tubes of differently fluorescently labeled monoclonal antibodies were prepared per each donor. Samples from different BMI groups were always stained and measured simultaneously as a set from thawed PBMCs over the course of several weeks. A backbone staining with the antibodies CD3, CD14, CD16, and CD56 was used in each panel tube to clearly distinguish the different immune cell populations and to identify specific surface molecules on NK cells and subsets.

For surface staining, 1 × 10^6^ PBMCs in 50 μl FACS buffer (PBS, 1% FBS, 2 mM EDTA, 0.05% sodium azide) per tube and donor were used. Non-specific FcR-binding was blocked by incubating FcR blocking reagent (Miltenyi Biotec, Bergisch Gladbach, Germany) followed by incubation with respective antibodies for 15 min at 4°C protected from light. All samples were stained with 7-AAD (7-amino-actinomycin D, Miltenyi Biotec) prior to analysis to exclude dead cells from downstream analysis.

#### Intracellular Staining and IFN-γ Detection

Freshly thawed PBMCs of blood donors were resuspended in cRPMI, cell numbers and cell viability were determined by AO (acridine orange)/PI (propidium iodide) staining (Logos Biosystem, Annandale, United States). Peripheral blood mononuclear cells were seeded (2 × 10^6^ cells/ml per well) in a 24-well plate and stimulated either with 10 ng/ml interleukin IL-12 (STEMCELL Technologies, Grenoble, France) in combination with 50 ng/ml IL-18 (InvivoGen, Toulouse, France) overnight at 37°C or with 50 ng/ml PMA (phorbol-12-myristate-13-acetate, InvivoGen, Toulouse, France) and 1 μg/ml ionomycin (Merck Chemicals, Darmstadt, Germany) for 4 h. Prior to the 4 h incubation, 10 μg/ml brefeldin A (Life Technologies, Darmstadt, Germany) was added to the cells to inhibit secretion of IFN-γ. Unstimulated PBMCs served as controls. After 4 h of stimulation, samples were washed, incubated with FcR blocking reagent and stained with the CD3, CD14, CD16, and CD56 (Supplementary Table 1) extracellular staining antibodies for 15 min at 4°C in the dark as backbone staining to identify NK cells in later analyses. Live/Dead Fixable Yellow was added to all sample tubes to allow discrimination between live and dead cells. After washing and fixation, cells were stained for intracellular markers using the inside stain kit (Miltenyi Biotec) according to manufacturer’s instructions. Peripheral blood mononuclear cells were intracellularly stained for 20 min at room temperature protected from light with the respective fluorochrome-conjugated antibodies (Supplementary Table 2). After washing with inside perm, cells were resuspended in 250 μl FACS buffer. Unstimulated PBMCs were also stained extra- and intracellularly and served as negative controls as well as for the determination of intracellular expression of EOMES (eomesodermin), T-bet (T-cell associated transcription factor), perforin, and Blimp-1 (B lymphocyte-induced maturation protein-1).

#### CD107a Degranulation Assay

CD107a expression on NK cells was measured to analyze NK cell degranulation. Peripheral blood mononuclear cells stimulated overnight with 10 ng/ml IL-12 and 50 ng/ml IL-18 or untreated PBMCs were washed and treated afterward either with K562 cells with an effector-to-target (E:T) ratio of 15:1 or without target cells for 4 h. Peripheral blood mononuclear cells stimulated only with PMA (50 ng/ml) plus ionomycin (1 μg/ml) served as positive controls. Prior to 4 h incubation with the CD107a-PE antibody (BD Biosciences, San Diego, CA, United States) monensin (5 μg/ml, BioLegend, Coblenz, Germany) and brefeldin A (5 μg/ml) were added. For every condition, CD107a-unstained controls were included as well as unstimulated samples with CD107a staining to detect spontaneous degranulation. After 4 h of incubation, samples were washed and extracellular backbone immunofluorescence staining was performed as described above (see section “Surface Staining and Determination of Extracellular Markers”).

#### Flow Cytometric Measurements and Gating Strategies

All samples were measured by using the flow cytometry analyzer BD LSRFortessa^TM^ (Becton Dickinson, Heidelberg, Germany). Measured data were analyzed using the BD FACS Diva^TM^ software version 7.0 (Becton Dickinson) or Flowlogic^TM^ version 700.2A (Miltenyi Biotec). For the immunophenotyping of NK cells by flow cytometric analysis 100,000 events were measured per panel. The NK cell phenotyping of extra- and intracellular markers was based on the gating strategies depicted in Supplementary Figures 1, 2. Data are presented as percentage or as median fluorescence intensity (MFI). Median fluorescence intensities were determined of all events in the respective channel or for the beforehand positive gated population. Both live and dead cell analyses were performed by exclusion of dead cells by the use of respective agents. Co-expression analysis were performed for some surface markers stained together in one flow cytometric multi-color panel. TriMap plots were generated with pre-cleared downsampled seven-plex multicolor data sets using FlowJo^TM^ (version 10.6.2) and relevant plugins.

### Multiplex Immunoassay

For analyses of cytokine secretion, the supernatants of the overnight incubated, unstimulated PBMCs of the degranulation assay was collected and stored at -80°C until analyzed. Measurements of cytokine concentrations were performed using a multiplex immunoassay (HCD8MAG-15K, Merck Chemicals) following the manufacturer’s instructions. Cytokine levels were determined using the BIO-PLEX 200 system (Biorad Laboratories, Feldkirchen, Germany) and the Bio-Plex Manager^TM^ software (Biorad Laboratories).

### NK Cell Purification and Real-Time Cytotoxicity Assay

Primary human NK cells were isolated from PBMCs by negative magnetic activated cell sorting (MACS) using a human NK Cell Isolation Kit (Miltenyi Biotec) according to manufacturer’s instructions. The purity of NK cells (85–90%) was checked on a sample basis by flow cytometric analysis. CD3^+^ T cell contamination in purified NK cells was <1%.

Cytotoxicity assays were performed using the real-time cell analysis (RTCA) systems instruments (iCELLigence, ACEA, Biosciences). The human target cells DLD-1 and MCF-7 were both seeded with a cell number of 40,000 cells/400 μl/well on the E-plates. On the following day, NK cells of one donor were isolated from PBMCs by MACS enrichment. After culturing DLD-1 and MCF-7 target cells for 24 h, the freshly isolated NK cells were added at an E:T ratio of 15:1 in a volume of 100 μl cRPMI in the dedicated wells. In addition, 200 U/ml hIL-2 was added in each well. One well of each E-plate was constantly loaded with medium only and served as a medium control as well as one well was always loaded with NK cells only. Wells with target cells alone served as untreated growth controls. After adding of NK cells, data were collected at defined time points over a period of 45 h. Each sample was usually analyzed in double or triple determination.

The cytolytic activity of the effector NK cells at a given time point was determined by using the calculated normalized cell index according to the instructions by the manufacturer.

### Statistical Analysis

Statistical analyses were performed using Graph Pad Prism software V6.07 (GraphPad, La Jolla, CA, United States). Identified outliers using GraphPad Prism’s Grubbs method were excluded from statistical analyses. To compare two groups (overweight or obese BMI groups were compared to normal weight BMI control group), Student’s *t* test (data were normally distributed with equal variances), the *t*-test with Welch’s correction (data were normally distributed with unequal variances) or the non-parametric Mann–Whitney *U* test (date were not normally distributed) were performed. One-way analysis of variance (ANOVA) with *post hoc* Tukey’s multiple comparison was used to compare the means of the normally distributed data of the human blood donor data (age, weight, height, BMI) of the three BMI groups. Human flow cytometric data are presented as box and whisker plots with median ± minimum to maximum values with additional dot plot representing individual donors. Real-time cell analysis results are presented as mean ± SEM (standard error of mean). Differences were considered as significantly different at *P* ≤ 0.05. *P* values are indicated as follows: ^∗^*P* ≤ 0.05, ^∗∗^*P* ≤ 0.01, and ^∗∗∗^*P* ≤ 0.001.

## Results

### Study Population

The study population was composed of 46 male subjects, which were divided into three groups according to the calculated BMI: normal weight (BMI 18.5–24.9 kg/m^2^, *n* = 14), overweight (BMI 25–29.9 kg/m^2^, *n* = 16) and obese (BMI ≥ 30 kg/m^2^, *n* = 16). The study subjects were aged between 19 and 66 years. No significant differences were observed in age, height and human cytomegalovirus (HCMV) serostatus between the three BMI groups. In result of their classification into the BMI groups, body weight and BMI were significantly different between normal weight, overweight and obese individuals (Table 1).

**TABLE 1:** Characteristics of the human study population.

	Normal weight Mean ± SEM (*n* = 14)	Overweight Mean ± SEM (*n* = 16)	Obese Mean ± SEM (*n* = 16)	One-way ANOVA *F* statistic (*P*-value)
Age (years)	37.7 ± 3.9	47.3 ± 3.6	49.6 ± 3.6	0.0980
Body height (cm)	182.9 ± 1.8	178.4 ± 1.1	178.8 ± 2.6	0.2251
Body weight (kg)	74.6**^*c*^** ± 1.9	87.5**^*b*^** ± 1.2	105.5**^*a*^** ± 4.3	**<0.0001**
BMI (kg⋅m^–2^)	22.3^*c*^ ± 0.4	27.5^*b*^ ± 0.3	32.8^*a*^ ± 0.5	**<0.0001**
Number of HCMV seronegative	5	4	5	0.8173^§^
Number of HCMV seropositive	9	12	11	

*Means with different letters are statistically significantly different according to *post hoc* Tukey’s multiple comparison test results. Bold *P*-values mark statistical significance. ANOVA, analysis of variance; BMI, body mass index; HCMV, human cytomegalovirus; SEM, standard error of the mean; ^§^ The one-way non-parametric ANOVA (Kruskal–Wallis test) was used to compare significant differences between the three BMI groups, including the respective seronegative (entered as 0) and seropositive (entered as 1) donors of each group.*

### Immune Cell Populations in Peripheral Blood Mononuclear Cells

**FIGURE 1:**
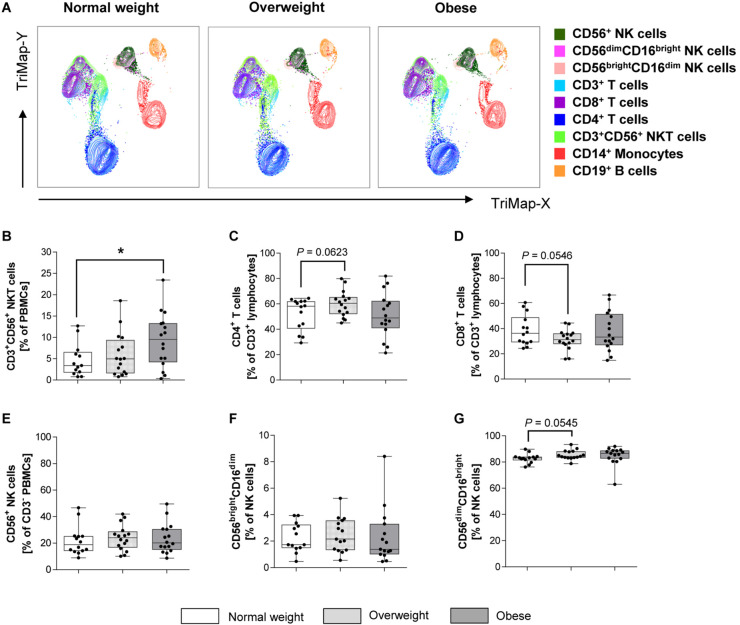
Frequencies of immune cell populations in peripheral blood mononuclear cells (PBMCs) isolated from normal weight (*n* = 14), overweight (*n* = 16), and obese (*n* = 16) healthy blood donors. **(A)** TriMap plots are presented showing the combined clusters of all donors subdivided into normal weight, overweight and obese. Clusters were identified by manual gating using the surface marker indicated on the legend. Percentage of CD3^+^CD56^+^ natural killer T (NKT) cells **(B)** within viable PBMCs. CD4^+^ helper T lymphocytes **(C)** and CD8^+^ cytotoxic T lymphocytes **(D)** as percentages of CD3^+^ T lymphocytes. Percentage of CD56^+^ NK cells in CD3^–^ cells **(E)**. Percentage of CD56^bright^CD16^dim^ NK cells **(E)** and CD56^dim^CD16^bright^ NK cells **(G)** in NK cells. **(B–G)** Graphs are box and whisker plots with median ± minimum to maximum value; with additional dot plot representing individual donors. Overweight and obese groups were compared to normal weight control group. Statistical significance is indicated as: *P ≤ 0.05; exact *P*-values within 0.05 ≤ *P* ≤ 0.1 are indicated.

Figure 1A displays TriMap plots showing dimensionality reduction on pre-cleared data for a 7-plex panel consisting of all marker illustrated in the legend. Using manual gating, no major differences for the main immune cell populations, except for changes in NKT cells were observed. CD3^+^CD56^+^ NKT cells were significantly increased in the obese subjects compared to the normal weights, whereas no significant difference was observed between overweight and normal weight groups (Figure 1B). No significant differences were detected in percentages of CD14^+^ monocytes, CD19^+^ B lymphocytes and total CD3^+^ T cells (data not shown). In addition, the percentages of the CD4^+^ and CD8^+^ T cell subsets did not differ between overweight or obese groups in comparison to normal weight donors (Figures 1C,D).

The CD3^–^CD56^+^ NK cell percentages in PBMCs, in the following referred to as total NK cells, as well as the percentage of CD56^bright^CD16^dim^ and CD56^dim^CD16^bright^ NK cell subset in total NK cells did not significantly differ between BMI groups (Figures 1E–G).

### Expression of Killer-Cell Immunoglobulin-Like Receptors (KIRs) on NK Cells

**FIGURE 2:**
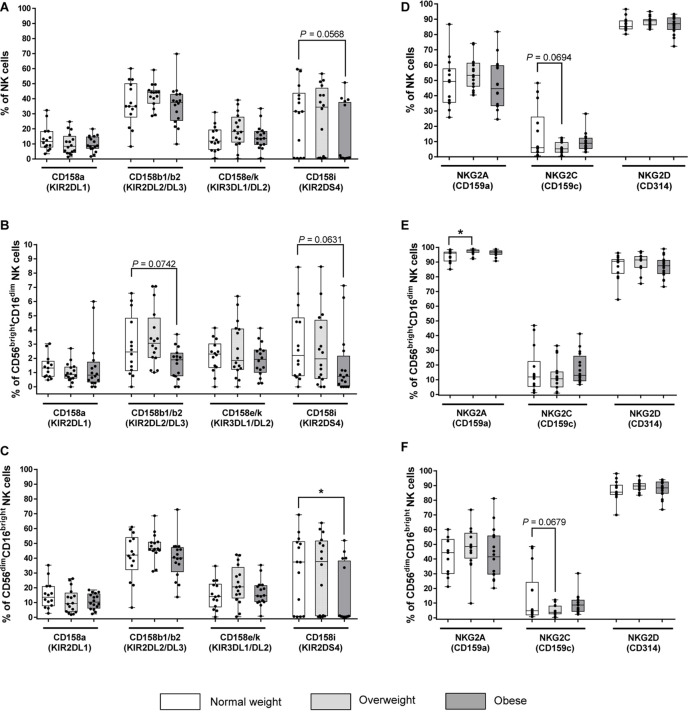
Frequencies of NK cells and NK cell subsets expressing killer-cell immunoglobulin-like receptors (KIRs, **A–C**) and C-type lectin receptors (CTLRs, **D–F**) in peripheral blood mononuclear cells (PBMCs) isolated from normal weight (*n* = 14), overweight (n = 16) and obese (n = 16) healthy blood donors. **(A–C)** Percentage of KIR^+^ total NK cells **(A)**, CD56^bright^CD16^dim^ NK cells **(B)**, and CD56^dim^CD16^bright^ NK cells **(C)**. KIRs named on X-axes are inhibitory (CD158a, CD158b1/b2, CD158e/k) and activating (CD158i) receptors on NK cells. **(D–F)** Percentage of CLR^+^ total NK cells **(D)**, CD56^bright^CD16^dim^ NK cells **(E)**, and CD56^dim^CD16^bright^ NK cells **(F)**. CTLRs named on X-axes are inhibitory (NKG2A) and activating (NKG2C, NKG2D) receptors on NK cells. Graphs are box and whisker plots with median ± minimum to maximum value; with additional dot plot representing individual donors. Overweight and obese groups were compared to normal weight control groups. Statistical significance is indicated as: *P ≤ 0.05; exact *P*-values within 0.05 ≤ *P* ≤ 0.1 are indicated.

Across different markers, KIR expression is 10 to 20 times higher in CD56^dim^CD16^bright^ NK cells as compared to CD56^bright^CD16^dim^ NK cells, but independent of BMI (Figures 2B,C). No significant changes of the expression of the inhibitory KIRs KIR2DL1, KIR2DL2/DL3 and KIR3DL1/DL2 on total NK cells or NK cell subsets were detected between different BMI groups (Figures 2A–C). Interestingly, the expression of the activating KIR2DS4 on CD56^dim^CD16^bright^ NK cells was highly significantly decreased in obese compared to normal weight subjects, whereas no significant differences were observed in total and CD56^bright^CD16^dim^ NK cells (Figures 2A–C).

### Expression of C-Type Lectin-Like NKG2 Receptors on NK Cells

Among all BMI groups, the proportion of total NK cells and CD56^dim^CD16^bright^ NK cells expressing the activating NKG2D receptor was the highest, while the proportion of the same populations expressing the inhibitory NKG2A receptor was almost 50% lower (Figures 2D,F). In contrast, the proportions of NKG2D^+^ and NKG2A^+^CD56^bright^CD16^dim^ NK cells were almost the same with mainly over 80% positive cells among all BMI groups. Moreover, the proportion of NKG2C^+^ cells was the lowest with less than 17% positive cells in total NK cells as well as in both NK cells subsets of all BMI groups (Figures 2D–F). The proportion of NKG2A^+^ cells was equal in total and CD56^dim^CD16^bright^ NK cells, but it was significantly higher in overweight individuals compared to normal weight individuals in CD56^bright^CD16^dim^ NK cells (Figures 2D–F). No significant changes of NKG2C^+^ cells and NKG2D^+^ cells were observed in total NK cells and NK cell subsets between the BMI groups (Figures 2D–F).

### Expression of Natural Cytotoxicity Receptors on NK Cells

**FIGURE 3:**
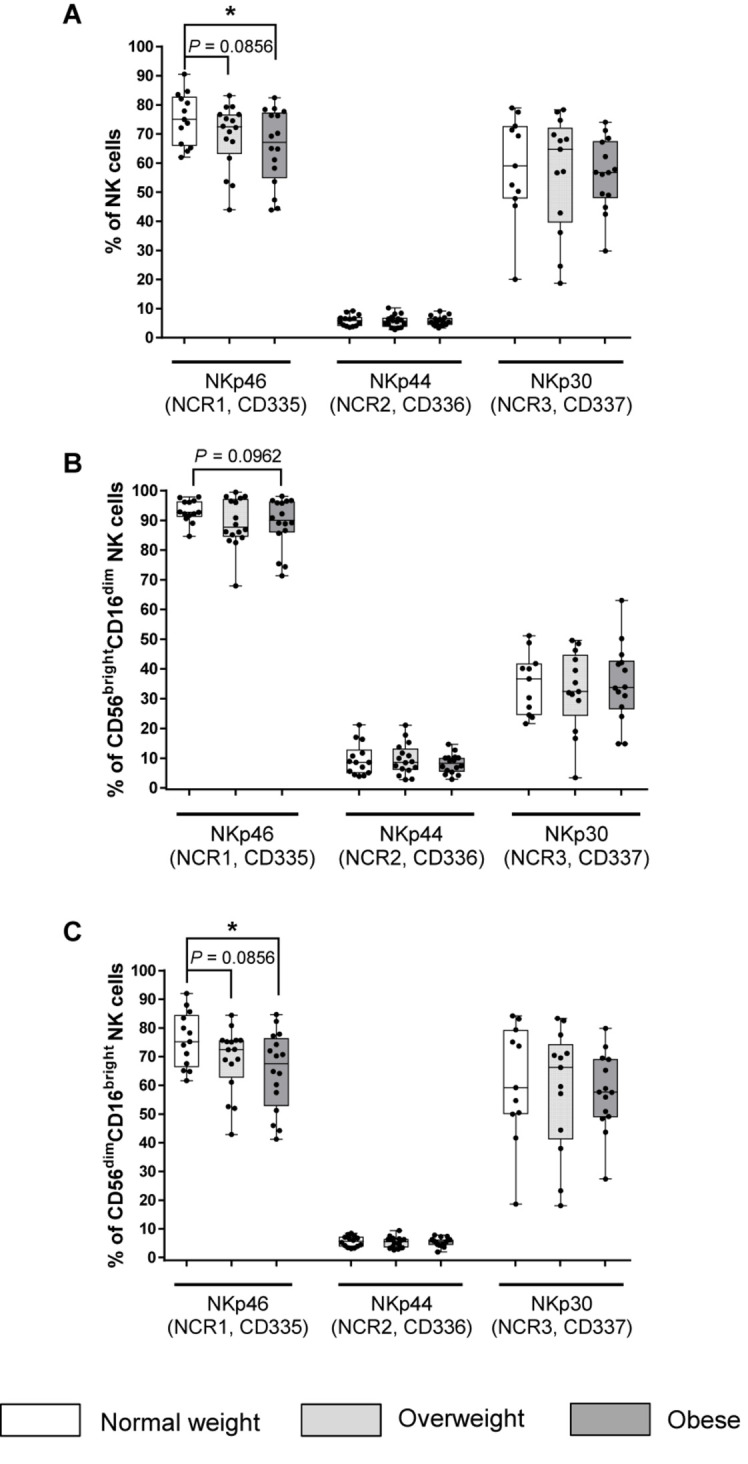
Frequencies of NK cells and NK cell subsets expressing natural cytotoxicity receptors (NCRs) in peripheral blood mononuclear cells (PBMCs) isolated from normal weight (*n* = 11–14), overweight (*n* = 13–16), and obese (*n* = 14–16) healthy blood donors. Percentage of NCR^+^ total NK cells **(A)**, CD56^bright^CD16^dim^ NK cells **(B)**, and CD56^dim^CD16^bright^ NK cells **(C)**. Graphs are box and whisker plots with median ± minimum to maximum value; with additional dot plot representing individual donors. Overweight and obese groups were compared to normal weight control groups. Statistical significance is indicated as: *P ≤ 0.05; *P*-values within 0.05 ≤ *P* ≤ 0.1 are indicated with precise *P*-values.

The expression of the activating receptor NKp46 was highest among the three NCRs on CD56^bright^CD16^dim^, CD56^dim^CD16^bright^ and total NK cells, whereas the proportion of activating receptor NKp44^+^CD56^bright^CD16^dim^, CD56^dim^CD16^bright^ and total NK cells was lowest, independent from the BMI group (Figures 3A–C). Obese individuals showed significantly decreased percentages of NKp46^+^ total and CD56^dim^CD16^bright^ NK cells compared to normal weight individuals (Figures 3A,C). No significant differences were detected between BMI groups and the level of NKp44^+^ and NKp30^+^CD56^bright^CD16^dim^, CD56^dim^CD16^bright^ and total NK cells (Figures 3A–C).

### Expression of Other Inhibitory Receptors on NK Cells

The expression analysis of the inhibitory receptor CD161 revealed similar frequencies of CD56^bright^CD16^dim^, CD56^dim^CD16^bright^ and total NK cells bearing this receptor. No significant differences were found between BMI groups within the investigated NK cell populations or MFI analysis of the level of CD161 expression (Supplementary Figures 3A–C).

**FIGURE 4:**
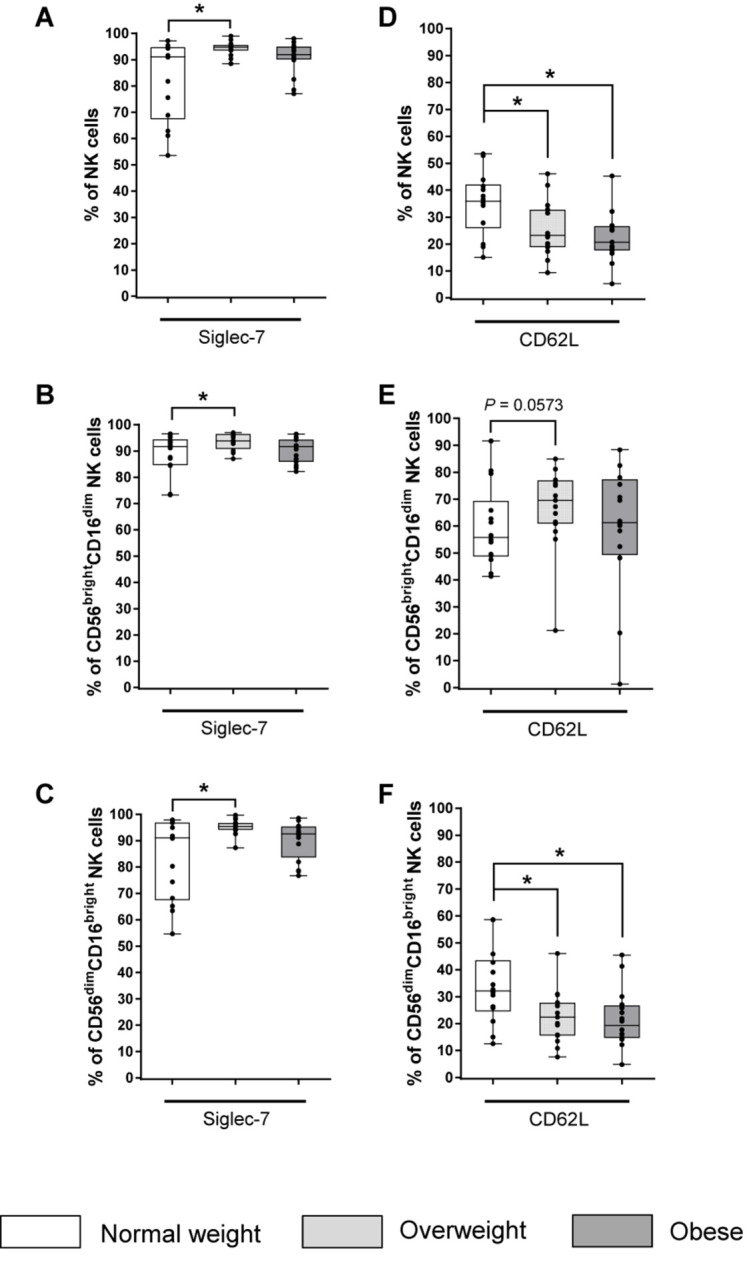
Frequencies of NK cells and NK cell subsets expressing the inhibitory receptor Siglec-7 **(A–C)** and the adhesion molecule CD62L **(D–F)** in peripheral blood mononuclear cells (PBMCs) isolated from normal weight (*n* = 14), overweight (*n* = 16), and obese (*n* = 16) healthy blood donors. Percentage of Siglec-7^+^ total NK cells **(A)**, CD56^bright^CD16^dim^ NK cells **(B)**, and CD56^dim^CD16^bright^ NK cells **(C)**. Percentages of CD62L^+^ total NK cells **(D)**, CD56^bright^CD16^dim^ NK cells **(E)**, and CD56^dim^CD16^bright^ NK cells **(F)**. Graphs are box and whisker plots with median ± minimum to maximum value; with additional dot plot representing individual donors. Overweight and obese groups were compared to normal weight control group. Statistical significance is indicated as: *P ≤ 0.05; *P*-value within 0.05 ≤ *P* ≤ 0.1 is indicated with precise *P*-value.

The majority of total NK cells and both NK cell subsets expressed the inhibitory receptor Siglec-7 (Figures 4D–F). Interestingly, overweight individuals showed increased frequencies of Siglec-7 expressing total NK cells as well as CD56^bright^CD16^dim^ and CD56^dim^CD16^bright^ NK cells compared to normal weight individuals (Figures 4A–C). However, no BMI-related effects were observed in the frequency of Siglec-7 on total NK cells or on NK cell subsets in obese compared to normal weight subjects (Figures 4A–C).

### Expression of Adhesion Molecules on NK Cells

The proportion of total and CD56^dim^CD16^bright^ NK cells expressing the adhesion molecule CD62L was significantly reduced in the obese and overweight groups compared to the normal weight group (Figures 4D,F).

No significant differences in frequencies and MFI have been revealed within total NK cells and the investigated NK cell subsets expressing the adhesion molecule CD2 comparing the three BMI groups (Supplementary Figures 3D–F).

The expression of the adhesion molecule DNAM-1 was nearly 100% on all investigated NK cell populations in all BMI groups (data not shown). No significant differences in MFI were observed between the BMI groups (Supplementary Figures 3G–I).

### Expression of Additional Functional Markers on NK Cells

To analyze the surface expression of activation-associated receptors, the percentage of CD25 and the percentage and MFI of CD69 on NK cells and NK cells subsets were determined. Analyses of the activation-associated receptor CD25 demonstrated no significant differences in the frequency of total NK cells and NK cells subsets expressing CD25 (Supplementary Figures 4A–C). In addition, no significant differences have been observed analyzing the MFI of CD69 expression in total NK cells as well as the two NK cell subsets (Supplementary Figures 4D–F).

**FIGURE 5:**
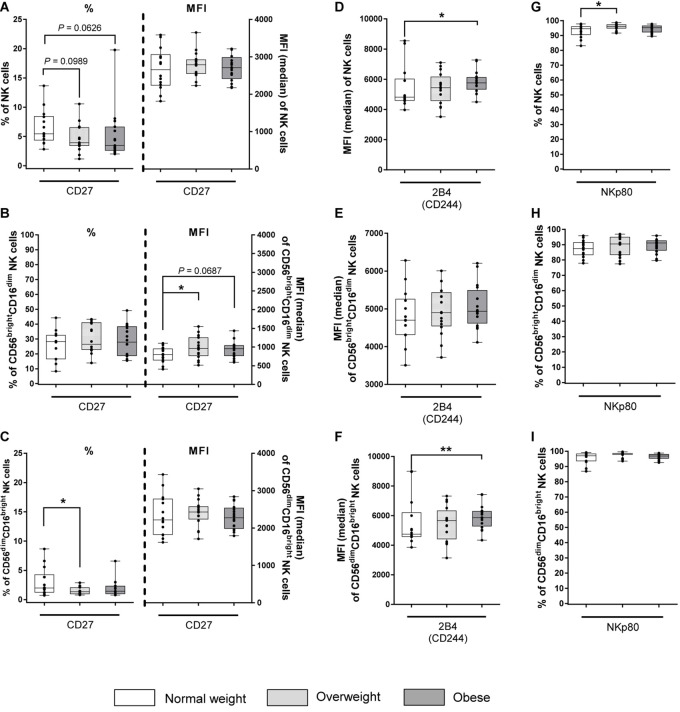
Frequencies and median fluorescent intensities (MFIs) of NK cells and NK cell subsets expressing the maturation and differentiation marker CD27 and the co-activating markers 2B4 and NKp80 in peripheral blood mononuclear cells (PBMCs) isolated from normal weight (*n* = 14), overweight (*n* = 16), and obese (*n* = 16) healthy blood donors. Percentage and MFI of CD27^+^ total NK cells **(A)**, CD56^bright^CD16^dim^ NK cells **(B)**, and CD56^dim^CD16^bright^ NK cells **(C)**. MFI of 2B4^+^ total NK cells **(D)**, CD56^bright^CD16^dim^ NK cells **(E)**, and CD56^dim^CD16^bright^ NK cells **(F)**. Percentage of NKp80^+^ total NK cells **(G)**, CD56^bright^CD16^dim^ NK cells **(H)**, and CD56^dim^CD16^bright^ NK cells **(I)**. Graphs are box and whisker plots with median ± minimum to maximum value; with additional dot plot representing individual donors. Overweight and obese groups were compared to normal weight control group. Statistical significance is indicated as: *P ≤ 0.05; **P ≤ 0.01; *P* values within 0.05 ≤ *P* ≤ 0.1 are indicated with precise *P*-values.

The expression of CD27 and CD57 was investigated to analyze maturation and differentiation markers on total NK cells and NK cells subsets. The proportion of CD27^+^ cells was five to six times higher in CD56^bright^CD16^dim^ NK cells compared to total and CD56^dim^CD16^bright^ NK cells, respectively. In contrast, the MFI was lower in CD56^bright^CD16^dim^ NK cells compared to total and CD56^dim^CD16^bright^ NK cells (Figures 5A–C). No significant differences were detected between BMI groups in the proportion of CD27^+^ total and CD56^bright^CD16^dim^ NK cells (Figures 5A,B). However, analyses of the MFI of CD56^bright^CD16^dim^ NK cells revealed an increase for CD27 in the overweight group compared to the normal weight group (Figure 5B). Additionally, a significantly diminished frequency of CD56^dim^CD16^bright^ NK cells bearing the CD27 surface marker was observed in the overweight group compared to the normal weight group, without differences in the MFIs (Figure 5C). The proportion of CD57^+^ cells was eight times lower in CD56^bright^CD16^dim^ NK cells compared to total and CD56^dim^CD16^bright^ NK cells (Supplementary Figures 4G–I). In addition, the MFIs of CD57 was 25 times lower in CD56^bright^CD16^dim^ NK cells compared to total and CD56^dim^CD16^bright^ NK cells. No differences in the proportions and MFIs of the investigated CD57^+^ NK cell populations were detected between the BMI groups (Supplementary Figures 4G–I).

To characterize the surface expression of co-activating markers, the expression of 2B4 and NKp80 on total NK cells and NK cells subsets was analyzed. 2B4 expression was nearly 100% on all investigated NK cell populations in all BMI groups (data not shown). MFI of 2B4 was significantly enhanced in total NK cells and CD56^dim^CD16^bright^ NK cells in obese subjects compared to normal weight subjects, whereas equal MFIs for 2B4 were detected for CD56^bright^CD16^dim^ NK cells across different BMI groups (Figures 5D–F). For NKp80, the total NK cell population as well as both NK cell subsets displayed >80% positive cells regardless of the BMI. However, a significantly increased frequency of NKp80^+^ total NK cells in overweight subjects compared to normal weight subjects was detected, whereas no BMI-related differences were observed in both NK cells subsets (Figures 5G–I).

In addition to investigations on co-activating markers, the expression of the inhibitory co-signaling receptor TIGIT and the inhibitory co-receptor PD-1 was analyzed. The frequencies and MFI of TIGIT and PD-1^+^ NK cells within all investigated NK cell populations revealed no differences between total NK cells and NK cell subsets or between the three BMI groups (Supplementary Figure 5).

**FIGURE 6:**
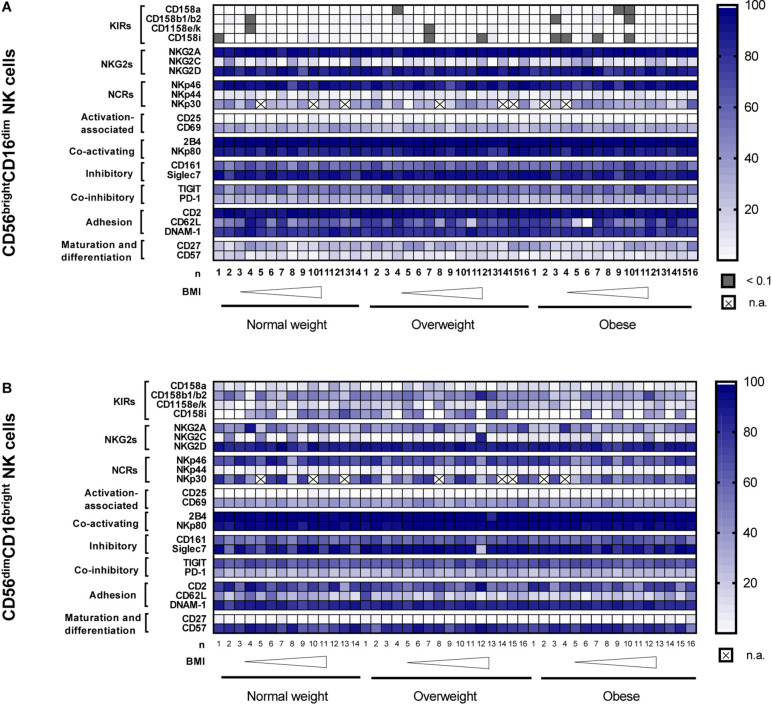
Heatmaps showing the surface receptor expression on CD56^bright^CD16^dim^ NK cells **(A)** and CD56^dim^CD16^bright^ NK cells **(B)** as frequencies of total NK cells. Data were analyzed in peripheral blood mononuclear cells (PBMCs) isolated from normal weight (*n* = 11–14), overweight (*n* = 14–16) and obese (*n* = 14–16) healthy blood donors. The donors are ordered by increasing body mass index (BMI) from left to right. CD, cluster of differentiation; BMI, body mass index; DNAM-1, DNAX accessory molecule 1; KIRs, killer-cell immunoglobulin-like receptors; n.a., not analyzed; NCRs, natural cytotoxicity receptors; NKG2s, natural killer group 2 receptors; TIGIT, T-cell immunoreceptor with Ig and ITIM domains.

In Figure 6, the heatmaps summarize the surface receptor expressions on the CD56^bright^CD16^dim^ and CD56^dim^CD16^bright^ NK cell subsets as frequencies of total NK cells in normal weight, overweight and obese subjects ordered by the increasing BMI.

### Co-expression Analysis of Different NK Surface Markers

As co-expression of NK cell receptors has been associated with inflammatory states, we analyzed co-expression of different markers on NK cells of study subjects.

As the number of events of co-expressing CD56^bright^CD16^dim^ NK cell subset was too low for statistical evaluation, analysis of co-expression was performed only for total NK cells and the CD56^dim^CD16^bright^ NK cell subset. Supplementary Figure 6 displays the frequencies of total NK cells and CD56^dim^CD16^bright^ NK cells co-expressing different variations of interesting NK cell receptors. The frequencies of total NK cells and CD56^dim^CD16^bright^ NK cells that are double-positive for CD158a^+^CD158b1/b2^+^, CD62L^+^CD158e/k^+^, CD57^+^NKG2A^+^, CD57^+^NKG2C^+^ and NKG2A^+^NKG2C^+^ did not differ between the BMI groups (Supplementary Figures 6A–J).

### Analyses of Intracellular Maturation and Differentiation Markers

Intracellular staining of unstimulated PBMCs revealed no differences in the intracellular maturation and differentiation markers EOMES, T-bet and Blimp-1, neither in total and CD56^bright^ NK cells nor among the BMI groups (Supplementary Figures 7A,B).

### Cytolytic Activity of Primary Human NK Cells Against DLD-1 and MCF-7 Cells

To investigate a possible BMI-dependent impact on human NK cell killing capacity, cytotoxicity assays were performed using freshly isolated NK cells from PBMCs of blood donors with different BMIs and DLD-1 and MCF-7 cells as target cells.

**FIGURE 7:**
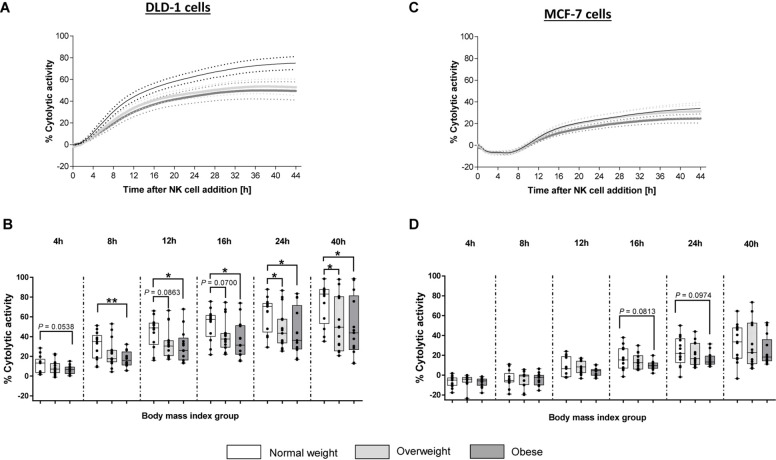
Cytolytic activity of primary human natural killer cells, isolated from blood donors with different body mass indexes [normal weight ([*n* = 13), overweight (*n* = 15), and obese (*n* = 13)], against the human colon carcinoma cell line DLD-1 or the human mamma carcinoma cell line MCF-7. Target cells were seeded with a density of 40,000 cells/well. After 24 h freshly isolated human NK cells were added at 15:1 E:T ratio. Impedance was measured at well-bottoms every 15 min for 45 h. Changes in impedance were given as dimensionless cell index (CI). Targets cells alone served as control. Based on CI values of target cells and target cells treated with NK cells, the percentage of cytolytic activity was calculated. **(A,C)** Total trend of time dependent cytolytic activity after NK cell addition. Data are expressed as mean ± SEM. **(B,D)** Specific cytolytic activity of primary human NK cells against DLD-1 **(B)** of MCF-7 **(D)** target cells at different time points after NK cell addition. Graphs are box and whisker plots with median ± minimum to maximum value; with additional dot plot representing individual donors. Overweight and obese groups were compared to normal weight control group. Statistical significance is indicated as: *P ≤ 0.05; **P ≤ 0.01; *P* values within 0.05 ≤ *P* ≤ 0.1 are indicated with precise *P*-values.

In cytotoxicity assays using DLD-1 target cells, no significant difference in the cytolytic NK cell activity was detected 4 h after NK cell addition. However, 8, 12, 16, 24, and 40 h after NK cell addition; the cytolysis of DLD-1 cells in the obese group was significantly reduced compared to the normal weight group (Figures 7A,B). In contrast to the obese group, the overweight group showed no significant differences in their cytolytic NK activity at 4, 8, 12, 16 h after NK cell addition compared to the normal weight group. However, at 24 and 40 h after NK cell addition, the cytolytic activity between the overweight group and the normal weight group was significantly decreased (Figures 7A,B).

In contrast to DLD-1 cells, results of cytotoxicity assays using MCF-7-1 target cells, showed no significant differences of cytolytic NK cell activity against the MCF-7 cells between the three BMI groups (Figures 7C,D). However, at 16 and 24 h post-NK cell-addition, a slight, but not significant, reduction in the cytolytic activity of NK cells derived from obese donors was determined compared to NK cells derived from normal weight donors (Figures 7C,D).

### Investigations on Killing Pathways of NK Cells

To investigate the killing pathways of NK cells to eliminate target cells, CD107a degranulation assay, measurements of intracellular expression of granzymes and perforin as well as intracellular TRAIL expression on NK cells of donors were performed.

**FIGURE 8:**
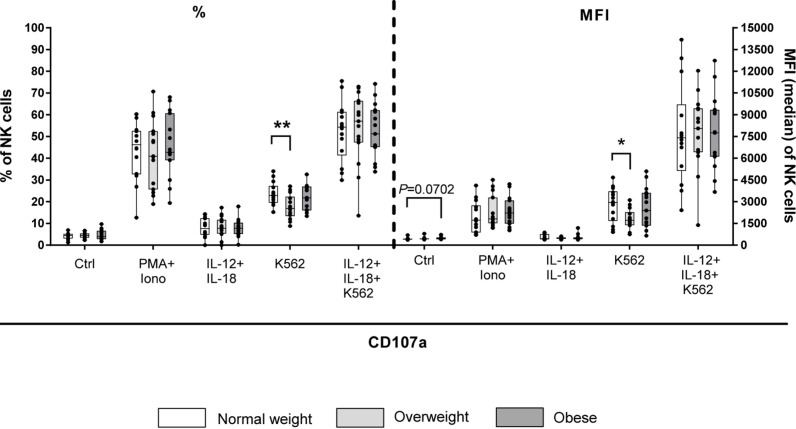
CD107a degranulation assay upon stimulation of PBMCs derived from blood donors with different body mass indexes [normal weight (*n* = 14), overweight (*n* = 15), and obese (*n* = 16)] either with 50 ng/ml phorbol-12-myristate-13-acetate (PMA) in combination with 1 μg/ml ionomycin for 4 h or with 10 ng/ml interleukin (IL)-12 in combination with 50 ng/ml IL-18 overnight or stimulation with K562 target cells at 15:1 effector to target ratio for 4 h or with the combination of IL-12 plus IL-18 overnight followed by K562 challenge prior to CD107a staining. Unstimulated samples with CD107a staining were included to detect spontaneous degranulation. Percentage and mean fluorescent intensity (MFI) of CD107a^+^ total NK cells are given. Graphs are box and whisker plots with median ± minimum to maximum value; with additional dot plot representing individual donors. Overweight and obese groups were compared to normal weight control group within each stimulation setting. Statistical significance is indicated as: *P ≤ 0.05; **P ≤ 0.01; *P* value within 0.05 ≤ *P* ≤ 0.1 is indicated with precise *P*-value. Ctrl: unstimulated control.

**FIGURE 9:**
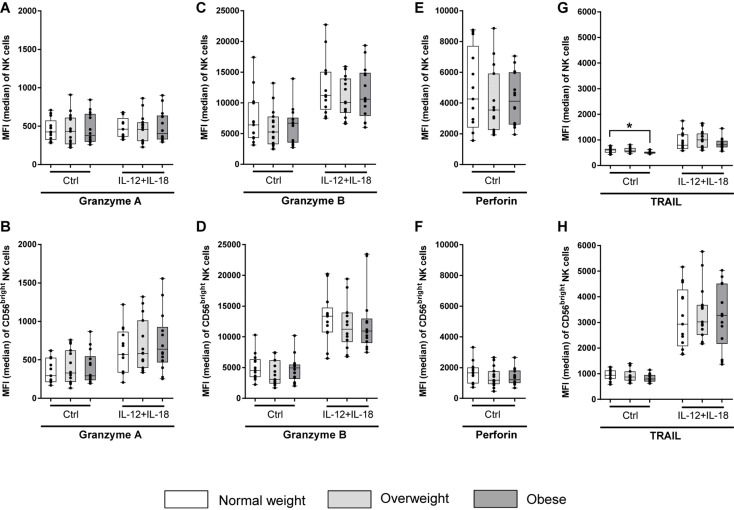
Median fluorescence intensity (MFI) of intracellular granzyme A, granzyme B, perforin, and tumor necrosis factor–related apoptosis-inducing ligand (TRAIL) in NK cells and CD56^bright^ NK cell subset. PBMCs of blood donors with different body mass indexes [normal weight (*n* = 13), overweight (*n* = 15), and obese (*n* = 14)] were either stimulated with 10 ng/ml interleukin (IL)-12 in combination with 50 ng/ml IL-18 overnight or remained untreated (perforin samples). **(A,B)** MFI of granzyme A^+^ total NK cells **(A)** and CD56^bright^ NK cells **(B)**. **(C,D)** MFI of granzyme B^+^ total NK cells **(C)** and CD56^bright^ NK cells **(D)**. **(E,F)** MFI of perforin^+^ total NK cells **(E)** and CD56^bright^ NK cells **(F)**. **(G,H)** MFI of TRAIL^+^ total NK cells **(G)** and CD56^bright^ NK cells **(H)**. Graphs are box and whisker plots with median ± minimum to maximum value; with additional dot plot representing individual donors. Overweight and obese groups were compared to normal weight control group. Statistical significance is indicated as: *P ≤ 0.05; Ctrl: unstimulated control.

Results of CD107a degranulation assay demonstrated that surface expression of CD107a was low on unstimulated NK cells (∼2%; Figure 8). Following stimulation with PMA plus ionomycin, CD107a surface expression on NK cells increased 20-fold, whereas stimulation with IL-12 plus IL-18 only led to a small increase in the frequency of CD107a^+^ NK cells. Upon stimulation with K562 target cells, frequencies of CD107a^+^ total NK cells of all BMI groups were about ten times higher compared to unstimulated cells. The combination of NK cell priming with IL-12 plus IL-18 overnight and K562 target cell challenge led to an increase in the frequency of total NK cells bearing CD107a on their surface; even higher than the stimulation with PMA plus ionomycin alone. Interestingly, K562 stimulation led to a significant decrease in frequency and MFI of CD107a^+^ total NK cells in overweight compared to normal weight individuals (Figure 8). Moreover, the expression of the intracellular NK cell markers granzyme A, granzyme B and perforin was assessed on unstimulated as well as IL-12 plus IL-18 stimulated total NK and CD56^bright^ NK cells (Figures 9A–F). The MFIs of granzyme A and granzyme B were nearly equal between total NK cells and CD56^bright^ NK cells. No significant differences were detected comparing the MFIs of granzyme A and granzyme B among the three BMI groups (Figures 9A–D). Analyses of the perforin expression demonstrated that the MFI value of perforin^+^CD56^bright^ NK cells was two to four times lower compared to total NK cells, without differences among the BMI groups (Figures 9E,F).

In addition, intracellular TRAIL expression was determined in NK cells of donors to investigate death receptor-mediated killing mechanisms. The MFI of stimulated TRAIL expressing CD56^bright^ NK cells was three times higher than of total NK cells among all BMI groups (Figures 9G,H). Interestingly, the MFI of TRAIL^+^ unstimulated total NK cells of the obese group was significantly lower compared to the normal weight group, whereas no BMI-related differences in the MFI of TRAIL was detected in CD56^bright^ NK cells or interleukin-stimulated total or CD56^bright^ NK cells (Figures 9G,H).

### Analyses of Cytokine and Chemokine Secretion of NK Cells and Peripheral Blood Mononuclear Cells

In order to investigate the potential of NK cell populations to produce IFN-γ, PBMCs of donors were stimulated either with IL-12 plus IL-18 overnight or with PMA plus ionomycin for 4 h. Stimulation with either IL-12 plus IL-18 or PMA plus ionomycin promoted the IFN-γ production in NK cells, detectable by the increased percentage of IFN-γ^+^ total NK cells and CD56^bright^ NK cells among all BMI groups compared to basal levels (unstimulated PBMCs; Supplementary Figures 8A,B). CD56^bright^ NK cells stimulated with IL-12 plus IL-18 showed a higher potential to produce INF-γ in comparison to the total NK cell population among all BMI groups (>80% vs. 50–60% positive cells, Supplementary Figures 8A,B).

Neither in unstimulated nor in stimulated PBMCs, the frequency of IFN-y^+^ total NK cells and CD56^bright^ NK cells differed between the three BMI groups.

In addition to flow cytometric analyses of IFN-γ production in NK cells, concentration of several cytokines and chemokines were measured in supernatants of the overnight incubated, unstimulated PBMCs of the degranulation assay. No significant differences regarding the levels of granulocyte-macrophage colony-stimulating factor (GM-CSF), IFN-γ, IL-10, IL-6, granzyme A and B, Macrophage inflammatory proteins 1 (MIP-1)-α and -β, TNF-α, and perforin in the supernatants could be detected between the BMI groups (Supplementary Table 3).

## Discussion

The prevalence of obesity and overweight among adults and children is rapidly rising worldwide and displays a major health problem due to the adverse health effects and costs, affecting both industrialized and developing countries (1). Excess body fat increases the susceptibility to infections and the risk to develop certain types of malignancies, including postmenopausal breast cancer and CRC (2, 57). Obese individuals are 40% more likely to die from cancer than non-obese individuals (58). Today, it is generally accepted that obesity causes immune dysregulation (59, 60). Nevertheless, the link between obesity and cancer susceptibility is still elusive. The altered phenotype and functionality of NK cells in obese individuals have been discussed to contribute to the increased cancer risk in obesity (22, 61). However, the available data on the meaning of functional NK cell receptors in dependence on body weight is insufficient. Moreover, studies with a focus on NK cell subsets in overweight and obese individuals have been rare.

Excluding donor criteria of the present study were acute infection, immunosuppression and malignant tumors. Therefore the herein presented results are unaffected by these conditions. Furthermore, age and CMV seropositivity may have effects on immune cell populations, e.g., T cells, B cells and NK cells (62–64). Our study groups were of similar age and equal CMV distribution, ensuring no influence of these parameters either. In the present study buffy coats derived from male blood donor were used, to gain first insights into the association of overweight and obesity with NK cell alterations, independent of hormonal fluctuations in women during the menstrual cycle on immune cells and especially the NK cells (65, 66).

Besides specific analyses of NK cells and NK cell subsets, percentages of blood leukocyte populations from the total amount of PBMCs were determined in the present study. The results demonstrate a significant increase of NKT cells in obese subjects compared to subjects with normal weight. In accordance, an animal study showed an increase in the proportion of NKT cells in obese rats, which displayed a higher colorectal tumor burden (53).

As NKT cells have been shown to regulate intestinal microbiotic homeostasis by controlling bacterial colonization, altered NK cell numbers in obesity may be associated with gut dysbiosis and thereby influence inflammatory processes (67, 68). Future studies are necessary to evaluate which role NKT cells play in regulating tumor development in obese individuals. In this study, the total NK cell population as well as the CD56^bright^CD16^dim^ and CD56^dim^CD16^bright^ NK cell subsets showed no significant differences in their frequencies between the BMI groups. These results confirm the results of earlier studies in obese humans (69–71). In contrast, other human studies demonstrated differences in the frequency of total and/or CD56^dim^ and CD56^bright^ NK cell subsets comparing obese and normal weight individuals (72–75). Besides to differences in study population like age, gender, ethnicity and social status, these contrary results may be explained by a different number or BMI classes of subjects, by the use of different markers and methods to identify NK cells or various strategies for NK cell differentiation in CD56^bright^ and CD56^dim^ NK cell subsets (22). In this study, for the first time, a comprehensive screening was performed to investigate the association of the human body weight with the expression of various receptors and functional markers on total NK cells as well as on both NK cell subpopulations. KIRs represent one of the most important NK cell receptor families with inhibitory or activating function in NK cell responses. In accordance with results of previous studies, all four analyzed KIRs in this study were higher expressed on CD56^dim^CD16^bright^ NK cells than on CD56^bright^CD16^dim^ NK cells, independent of the BMI group (76, 77). Interestingly, obese individuals showed significantly or tendentiously reduced frequencies or even a complete lack of activating CD158i (KIR2DS4) receptor-positive cells in total NK cells and in both investigated NK cell subsets compared to individuals with normal weight. This indicates a lower activation status of NK cells and NK cell subsets in obese individuals, which may contribute to a higher susceptibility to infections and tumors. Moreover, KIRs are expressed differently depending on the individual haplotype. Humans with KIR haplotype A comprise a gene content of inhibitory genes-KIR2DL1, 2DL 3 and 3DL1 and 2DS4, as the single activating gene. In contrast, group B KIR haplotype encodes a variable but more activating KIR repertoire which normally does not include 2DS4 (78, 79). Interestingly, results of the BELFAST study showed that subjects of the KIR A haplotype show significantly higher body weights compared to KIR B haplotype (80). As there is a known KIR2DS4-deleted variant of the KIR A haplotype, it would be of high interest to investigate the KIR haplotype of our obese study group. Alternative explanations for the highly reduced KIR2DS4 expression could be DNS/genetic mutations or differences in posttranslational modifications, leading to a different protein expression with unaltered genetics.

In line with previous studies, the inhibitory receptor NKG2A was higher expressed on CD56^bright^CD16^dim^ NK cells compared to total or CD56^dim^CD16^bright^ NK cells (26). Interestingly, overweight subjects, but not obese subjects, showed a significantly higher expression of NKG2A on CD56^bright^CD16^dim^ NK cells in comparison to normal weight subjects in the present study. Aside of this, the expression of the activating counterpart NKG2C was slightly decreased on CD56^dim^CD16^bright^ and total NK cells in the overweight group compared to the normal weight group, although results were not statistically significant. As both, NKG2A and NKG2C, are involved in regulating antiviral and antitumor responses of NK cells, these data indicate a decreased potential of NK cells in overweight and obese humans to defend virus-infected or malignant cells (81–83). In accordance to previous studies, no significant differences in NKG2D expression were detected within total NK cells between the BMI groups (53, 75, 84–87). In contrast, other studies demonstrated altered NKG2D expression in obese individuals, although with conflicting results. *In vitro* studies reported that human NK-92 cells expressed lower NKG2D relative mRNA levels after incubation with high levels of leptin (53). Furthermore, diet-induced obese rats showed a significantly decreased mRNA expression of NKG2D in splenic tissues, that was accompanied with cancer development and metastases (53, 88). These discrepancies of results may be explained by species-dependent or methodological differences (measurement of mRNA expression versus protein expression).

The expression analysis of the activating NCRs NKp44 and NKp30 on the different NK cell populations revealed no significant differences among the BMI groups. However, a significantly reduced frequency of NKp46-expressing total and CD56^dim^CD16^bright^ NK cells was observed in obese subjects compared to normal weight subjects. This corresponds to results of previous animal and human studies describing a decreased NKp46 expression in circulating, splenic and uterine NK cells of obese individuals (53, 84, 86–88). Moreover, treatment of human NK-92 cells with the adipokine leptin led to a significantly reduced NKp46 mRNA expression (53). As leptin-treated NK-92 cells also showed a significantly less cytotoxicity against CRC cells than untreated NK-92 cells, it was discussed that a reduced expression of the activating NKp46 may be associated with an impaired NK cell response against tumor cells, thus increasing the risk to develop cancer (53).

Analyses of the frequencies of the inhibitory receptor Siglec-7 showed a comparable expression among the examined NK cell populations. In line with previous studies, the frequency of NK cells expressing Siglec-7 showed no differences comparing obese and normal weight individuals (71). However, this study showed a decreased MFI of Siglec-7 on CD56^bright^ NK cells comparing normal weight and obese subjects. Interestingly, data of the present study demonstrated a significantly increased expression of Siglec-7 on total NK cells and both NK cell subsets in overweight compared to normal weight subjects for the first time.

The activity of NK cells is regulated by the balanced expression of activating and inhibitory surface receptors triggering a cascade of events inside the cells. The decreased frequencies of the NK cells expressing the activating receptors KIR2DS4 and NKp46 and the increased frequency of NK cells expressing the inhibiting receptors NKG2A and Siglec-7 in overweight and obese subjects compared to normal weight subjects might contribute to the observed lower BMI-related NK cell activity.

Adhesion molecules, such as CD2, CD62L, and CD226 (DNAM-1), participate in interactions of NK cells with other immune cells and virus-infected or malignant transformed cells and thus play a crucial role in generating an effective NK cell-mediated immune response (89). As previously described, CD56^bright^CD16^dim^ NK cells displayed a higher expression of the adhesion molecules CD2 and CD62L compared to the CD56^dim^CD16^bright^ NK cells subset (90, 91). In the present study, the frequencies of total NK cells and CD56^dim^CD16^bright^ NK cells bearing CD62L on their surfaces were significantly reduced in the overweight and obese group compared to normal weight subjects. As the homing marker plays an important role in migration and tissue infiltration of NK cells, a decreased expression of CD62L on NK cells may explain the increased risk for infections and cancer in overweight and obese individuals (41, 42).

It has been suggested that CD27 can serve as an additional marker to discriminate human NK cell subsets, comparably to mouse NK cell subset discriminations (92). In accordance with results from other studies, present data showed lower CD27 expression of the CD56^dim^CD16^bright^ NK cell subset than of the CD56^bright^CD16^dim^ NK cell subset (25, 92). Furthermore, CD56^dim^CD16^bright^ NK cells of overweight subjects presented significantly decreased expression of CD27 compared to normal weight subjects. In line, Vossen et al. showed, that decreased CD27 expression on NK cells and specifically on CD56^dim^CD16^bright^ NK cells is associated with high cytolytic activity and decreased cytokine production (93). In contrast, in our study, the decreased proportions of CD27^+^CD56^dim^CD16^bright^ in the overweight subjects compared to normal weight subjects was associated with a lower NK cell cytotoxicity.

Previous studies demonstrated that the co-stimulatory factors 2B4 and NKp80 are involved in IFN-γ and TNF-α secretion as well as in activation of NK cell cytotoxicity (43, 44, 94, 95). According to previous studies of Viel et al., frequencies of 2B4 expression on examined NK cell populations were not altered in overweight or obesity (84). However, results of the present study demonstrated an upregulated expression in numbers of 2B4 molecules per cell on total NK cells as well as on CD56^dim^CD16^bright^ NK cells in obese subjects compared to the normal weight subjects for the first time. In addition, the increased proportion of total NK cells expressing NKp80 was significantly increased in overweight subjects compared to normal weight subjects. Thus, NK cells of overweight and obese individuals may have an improved ability to be activated via co-stimulation of activating NK cell receptors by 2B4 or NKp80.

A previous study reported that co-expression of NK cell receptors are associated with inflammatory states (96). Although obesity displays a chronic low-grade inflammation, the present study showed no BMI-related alterations in the frequencies of total NK cells and CD56^dim^CD16^bright^ NK cells that are double-positive for CD158a^+^CD158b1/b2^+^, CD62L^+^CD158e/k^+^, CD57^+^NKG2A^+^, CD57^+^NKG2C^+^, and NKG2A^+^NKG2C^+^. Previous animal and human studies already demonstrated a decreased NK cell cytotoxicity in obese individuals (74, 75, 97–100). Most of these previous studies on NK cell cytotoxicity in obese individuals were performed using the murine lymphoma cell line YAC-1 or the human myelogenous leukemia cell line K652 as target cells. Interestingly, Lamas et al. demonstrated that cytotoxic activity of NK cells can differ depending on the target cell (55). In accordance, data of the present real-time cytotoxicity assay using the same primary human NK cells as effector cells led to different results depending on the applied target cells - either the CRC cell line DLD-1 or the breast cancer cell line MCF-7. Using DLD-1 cells as target cells, led to a significantly reduced cytolytic activity of NK cells of overweight and obese donors compared to NK cells of normal weight donors. Moreover, previous data on leptin-incubated NK cells demonstrated a decreased NK cell cytotoxicity against DLD-1 cells after administration of the adipokine (53). In contrast, using MCF-7 cells as target cells, real-time cytotoxicity assay of the present study observed no significant differences in cytolytic activity of NK cells between overweight or obese subjects and normal weight subjects. However, a slightly, but not significantly, reduced cytolytic activity of NK cells isolated from obese blood donors against MCF-7 cells was detected at late time points (16 and 24 h after NK cell addition). In addition, results demonstrated a 50% lower lysis rate of NK cells against MCF-7 target cells compared to DLD-1 target cells. These discrepancies may be explained by low levels of MHC-I molecules on MCF-7 cells or rather the lack of MHC-I expression in DLD-1 cells (101, 102). In addition, the caspase-3 deficiency, the low level of Fas receptor and the resistance to TRAIL-induced apoptosis of MCF-7 cells may be causes for the reduced NK cell mediated lysis of MCF-7 cells compared to DLD-1 cells (103–105). In accordance to the real-time cytotoxicity assays, results of the CD107a degranulation assay showed a significantly decreased percentage and MFI of CD107a+ NK cells in K562-stimulated PBMCs of overweight compared to normal weight subjects, which is in line with a previous study (84).

Interestingly, a significant decreased number of intracellular TRAIL molecules in total NK cells were determined in the obese group of unstimulated NK cells compared to the normal weight group, which confirms previous results (70). In contrast, no BMI-related differences were observed for perforin and granzyme expression in total and CD56^bright^ NK cells. These results indicate that the reduced cytotoxicity of NK cells in obese subjects is induced by an impaired death cell receptor-dependent killing pathway in NK cells, but not by an altered capacity to degranulate perforins and granzymes. As the observed significant changes in NK cell phenotype and functional markers in overweight and obese individuals are rather slight, future studies are necessary to identify additional underlying mechanisms leading to the significantly decreased cytotoxicity.

In conclusion, our results revealed alterations of frequencies of immune cell populations and the expression of activating and inhibitory NK cell receptors, and other NK cell-related markers on total NK cells and NK cell subsets in overweight as well as obese individuals. Furthermore, the cytolytic NK cell activity against CRC cells was reduced in overweight and obese and individuals. Data led to the suggestion that this reduced killing capacity may be caused by an altered death receptor pathway in obesity. Results also showed that impaired NK cell physiology is not only restricted to obesity; even overweight subjects already display alterations of NK cells. These NK cell alterations and dysfunction might be an explanation for the higher cancer risk in obesity. Interestingly, animal studies have demonstrated that the NK cell phenotype is strongly influenced by the metabolic environment, as obese-related alterations could be normalized by NK cell transfer of obese rats into normal weight rats (106). In addition, body weight reduction of obese individuals by dietary intervention, physical activity or bariatric surgery can re-activate altered NK cell functionality (22, 100, 107, 108). Therefore, prevention of overweight and fat mass reduction in obesity by physical training or caloric restriction may attenuate the risk for obesity-associated cancer types by improving the NK cell function.

## Data Availability Statement

The raw data supporting the conclusions of this article will be made available by the authors, without undue reservation.

## Ethics Statement

The studies involving human participants were reviewed and approved by Ethics committee, Medical Faculty of Martin Luther University Halle-Wittenberg, Magdeburger Straße 16 06112 Halle (Saale), Germany. The patients/participants provided their written informed consent to participate in this study.

## Author Contributions

IB and JS planned and supervised the study with the help of HK. WN and AH collected the PBMCs and conducted the flow cytometry analyses under the supervision and guidance of DQ. DQ designed the flow cytometric set up and analyzed the workflow. WN and AH performed NK cell purification and the cytotoxicity assays under supervision of IB and JS. WN, JS, and IB were the major contributors in writing the manuscript. DQ and HK revised the manuscript critically. All authors contributed to the article and approved the submitted version.

## Conflict of Interest

The authors declare that the research was conducted in the absence of any commercial or financial relationships that could be construed as a potential conflict of interest.
