# Supplementary material for: Characterization of Surface Receptor Expression and Cytotoxicity of Human NK Cells and NK Cell Subsets in Overweight and Obese Humans
Source: Front Immunol. 2020 Sep 23;11:573200. doi: 10.3389/fimmu.2020.573200 (PMC7546782; doi:10.3389/fimmu.2020.573200)
Supplement: Supplementary file 1 [file Data_Sheet_1.PDF]

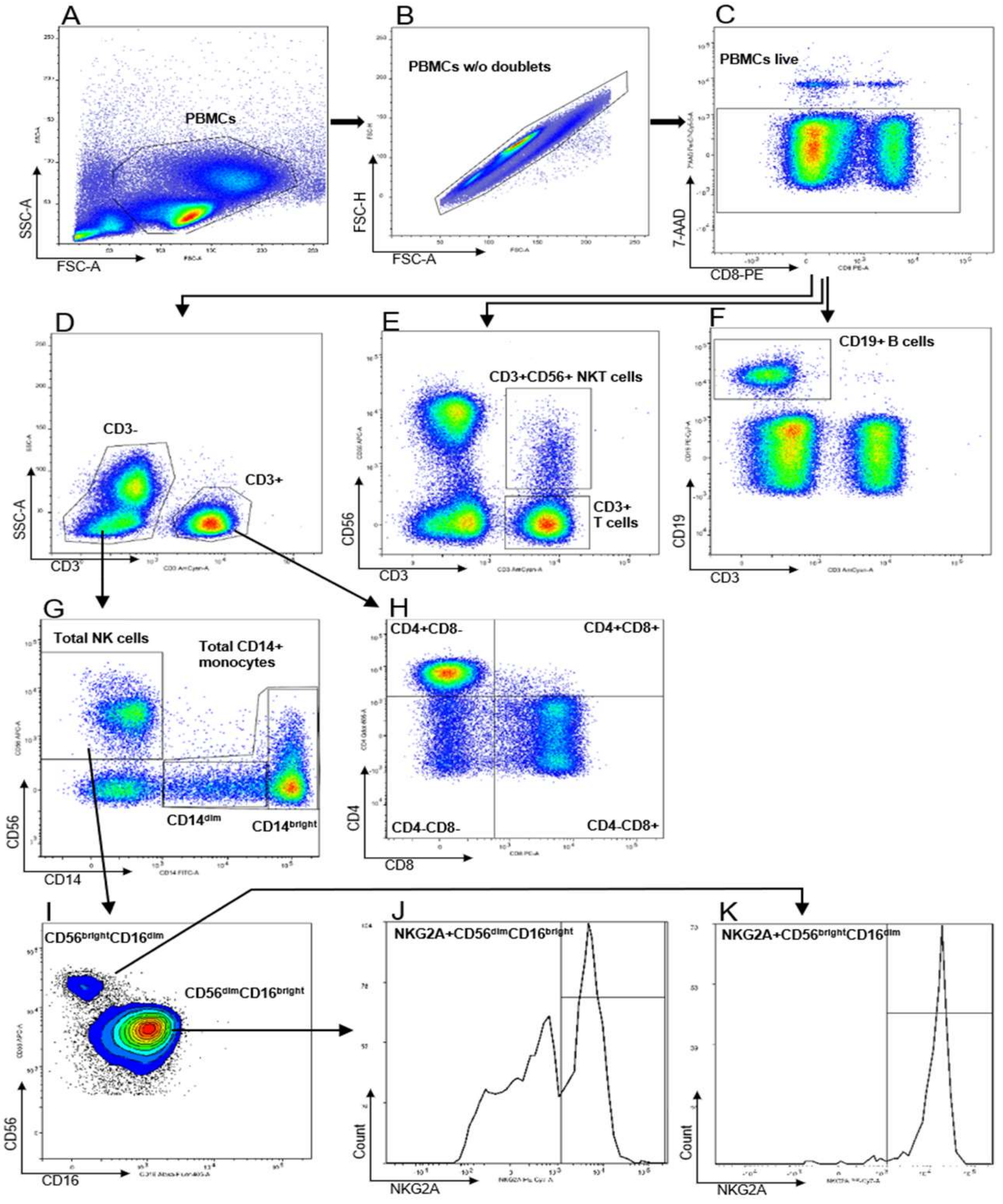

Supplementary Figure 1: Representative plots of the gating strategy to identify T cells, B cells, natural killer T (NKT) cells, monocytes and natural killer (NK) cells isolated from buffy coats of human blood donors. Lymphocytes were identified by their size and granularity in the FSC (forward-scattered light)/SSC (side-scattered light) dot plot (A), followed by doublet exclusion (B) and gating of viable cells (C). Based on the lymphocyte live gate, $CD3^+$ and $CD3^-$ cells (D), T cells ($CD3^+CD56^-$) and NKT cells ($CD3^+CD56^+$, E) as well as B cells ($CD19^+$, F) were classified. Monocytes ($CD14^+$) and NK cells ($CD56^+$) were selected from the $CD3^-$ fraction (G). $CD4^+$ T helper cells and $CD8^+$ cytotoxic T cells were selected from the $CD3^+$ fraction (H). On the basis of the expression of CD56 and CD16, the NK cell population was further identified and differentiated into the $CD56^{bright}CD16^{dim}$ and $CD56^{dim}CD16^{bright}$ NK cell subsets (I). The expression of each NK cell marker was assessed on both $CD56^{dim}CD16^{bright}$ (J) and $CD56^{bright}CD16^{dim}$ (K) NK cell subsets (here represented by histogram analysis of NKG2A expression for both subsets). Fluorescence minus one (FMO) control was used to determine positive staining.

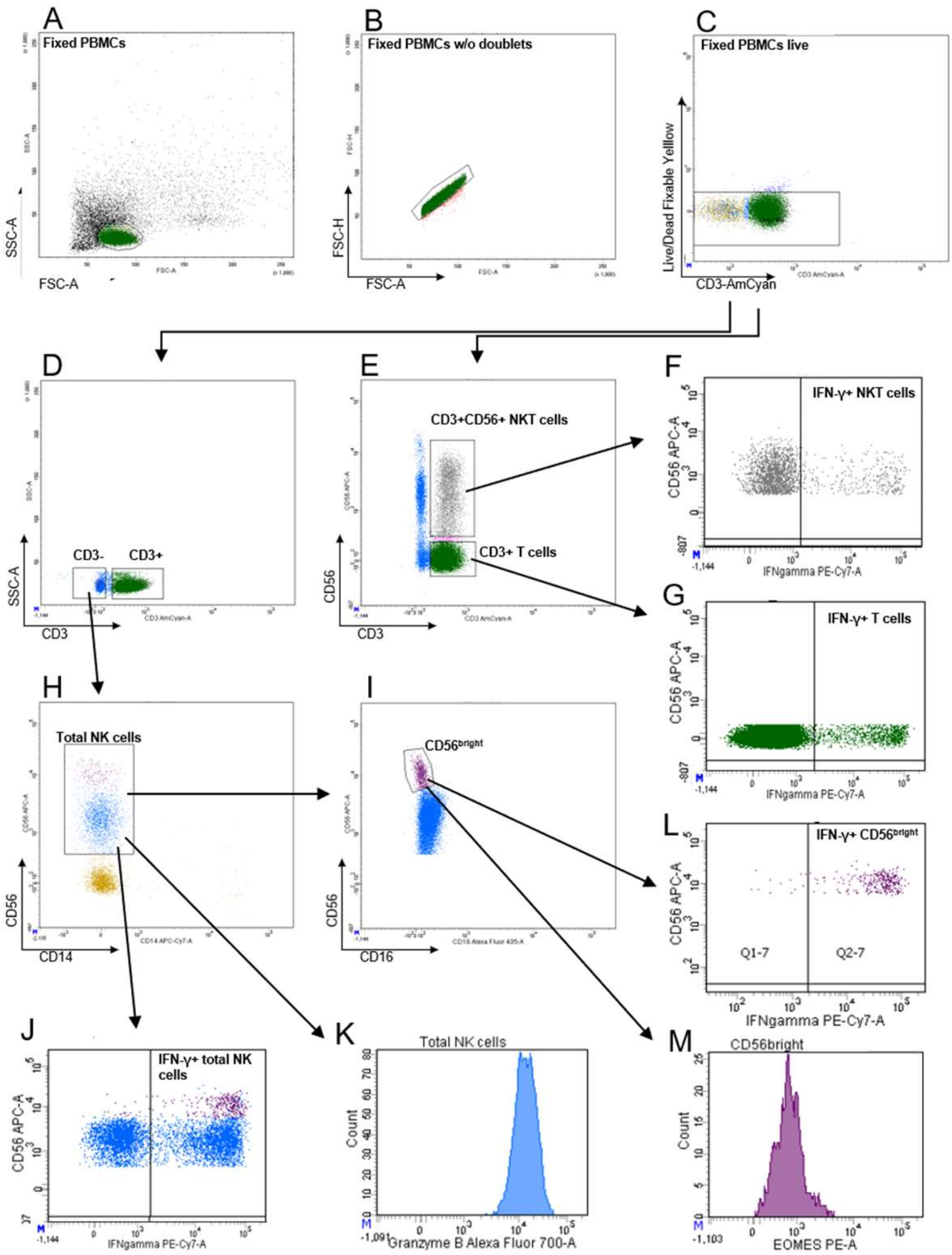

Supplementary Figure 2: Representative plots of the gating strategy to identify T cells, natural killer T (NKT) cells, and natural killer (NK) cells from buffy coats of human blood donors for the determination of intracellular markers. Lymphocytes were identified by their size and granularity in the FSC (forward-scattered light)/SSC (side-scattered light) dot plot (A), followed by doublet exclusion (B) and gating of viable cells (C). Based on the lymphocyte live fraction, cells were further divided into CD3$^-$ and CD3$^+$ cells (D), T cells (CD3$^+$CD56$^-$) and NKT cells (CD3$^+$CD56$^+$, E). Total NK cells were selected from the CD3$^-$ fraction (H) and further differentiated and identified into the CD56^{bright} NK cell subset. Representative density plots to demonstrate discrimination between positive and negative populations for the presence of intracellular interferon (IFN)-γ in NKT cells (F), T cells (G), total NK cells (J) and CD56^{bright} NK cells (L). Representative histograms of total NK cell (K) and CD56^{bright} NK cell subset (M) populations for histogram analysis to determine mean fluorescence intensity (MFI) of intracellular NK cell markers (here: represented by granzyme B and eomesodermin [EOMES]). FMO control was used to determine positive staining.

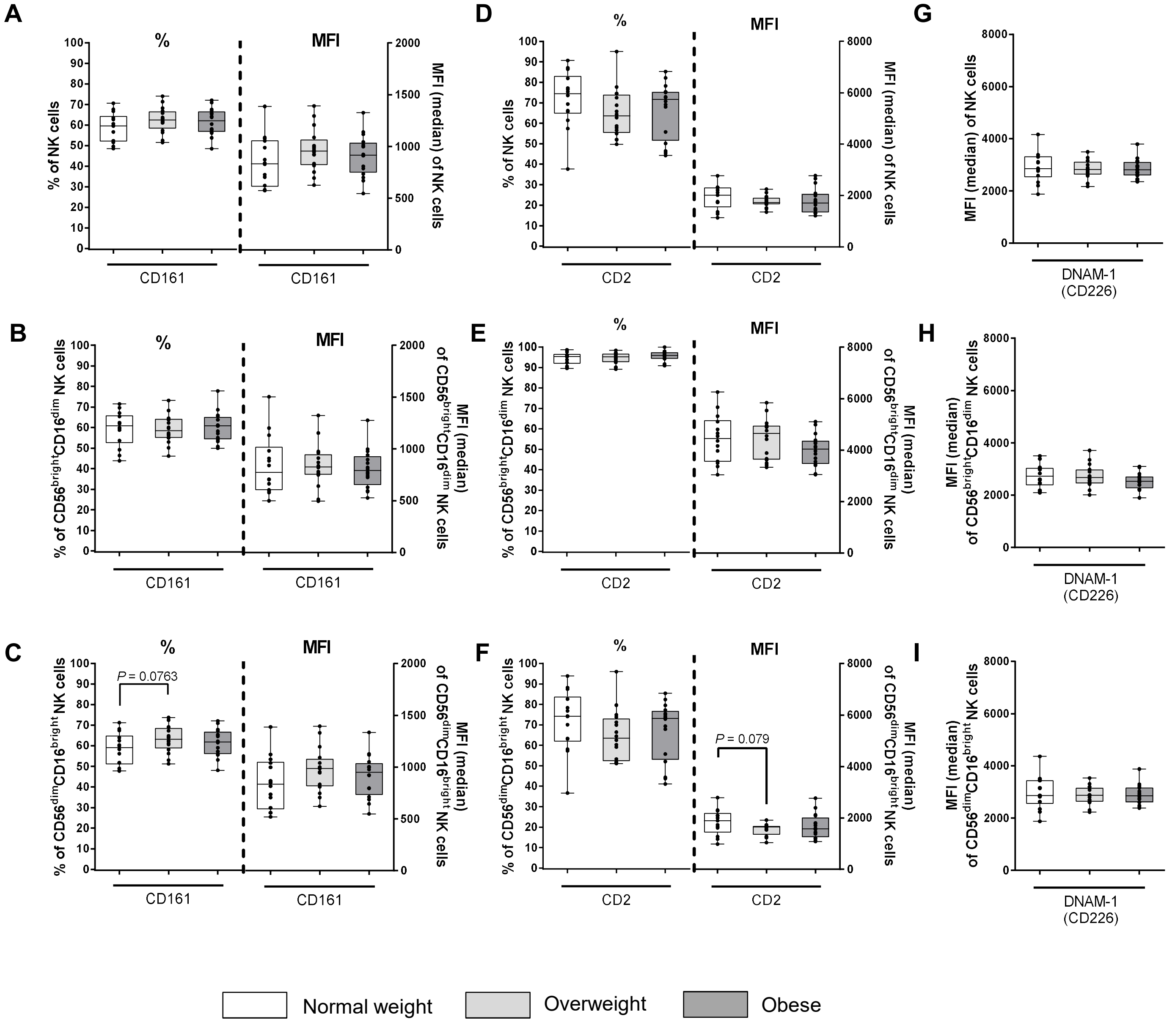

Supplementary Figure 3: Frequencies and median fluorescent intensities (MFIs) of NK cells and NK cell subsets expressing inhibitory receptors CD161 (A-C) and the adhesion molecules CD2 (D-F) and DNAM-1 (CD226, G-I), in peripheral blood mononuclear cells (PBMCs) isolated from normal weight (n=14), overweight (n=16) and obese (n=16) healthy blood donors. (A-C) Percentage and median of fluorescent intensity (MFI) of CD161^{+} total NK cells (A), CD56^{bright}CD16^{dim} NK cells (B) and CD56^{dim}CD16^{bright} NK cells (C). (D-F) Percentage of CD2^{+} total NK cells (D), CD56^{bright}CD16^{dim} NK cells (E) and CD56^{dim}CD16^{bright} NK cells (F). Percentage of DNAM-1^{+} total NK cells (G), CD56^{bright}CD16^{dim} NK cells (H) and CD56^{dim}CD16^{bright} NK cells (I). Graphs are box and whisker plots with median ± minimum to maximum value; with additional dot plot representing individual donors. Overweight and obese groups were compared to normal weight control group. Precise P-values within $0.05 < P < 0.1$ are indicated.

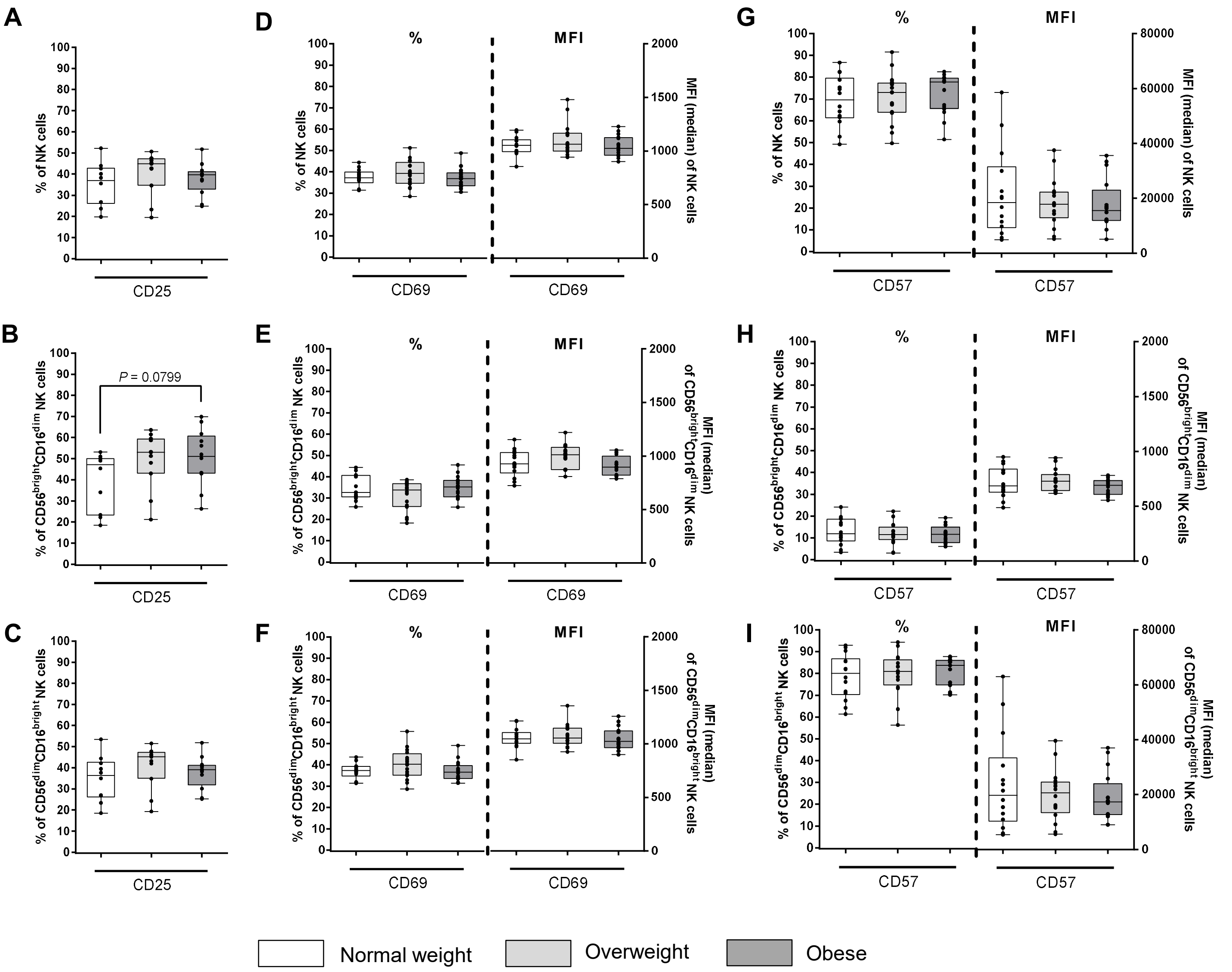

Supplementary Figure 4: Frequencies and median fluorescent intensities (MFIs) of NK cells and NK cell subsets expressing the activation-associated receptors CD25 (A-C) and CD69 (D-F) and the maturation and differentiation marker CD57 (G-I) in peripheral blood mononuclear cells (PBMCs) isolated from normal weight (n=14), overweight (n=16) and obese (n=16) healthy blood donors. Percentage of CD25$^+$ total NK cells (A), CD56^{bright}CD16^{dim} NK cells (B) and CD56^{dim}CD16^{bright} NK cells (C). Percentages and MFI of CD69$^+$ total NK cells (D), CD56^{bright}CD16^{dim} NK cells (E) and CD56^{dim}CD16^{bright} NK cells (F). Percentages and MFI of CD57$^+$ total NK cells (G), CD56^{bright}CD16^{dim} NK cells (H) and CD56^{dim}CD16^{bright} NK cells (I). Graphs are box and whisker plots with median ± minimum to maximum value; with additional dot plot representing individual donors. Overweight and obese groups were compared to normal weight control group.

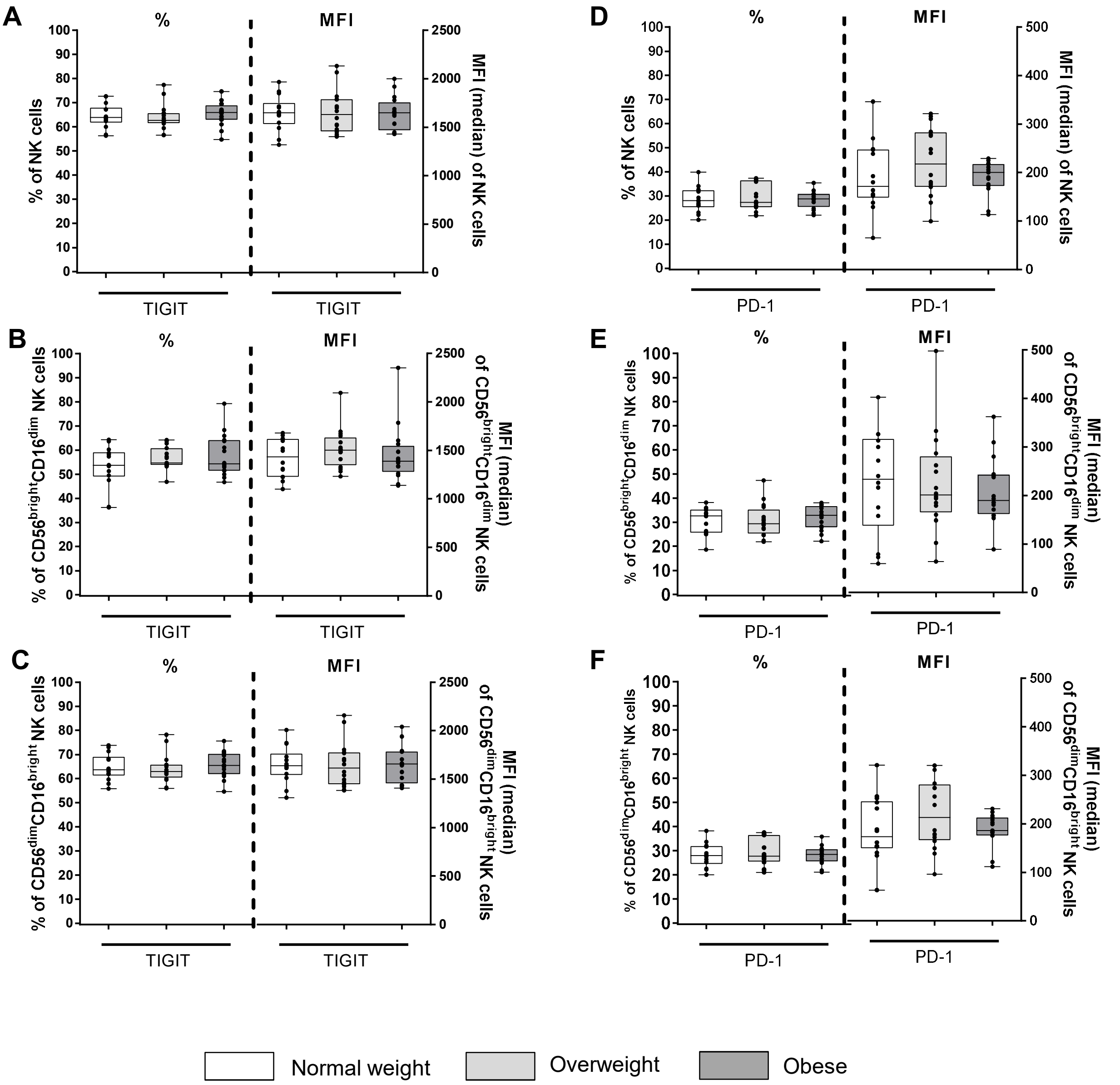

Supplementary Figure 5: Frequencies and median fluorescent intensities (MFIs) of NK cells and NK cell subsets expressing the co-inhibitory markers TIGIT (T-cell immunoreceptor with Ig and ITIM domains) and PD 1 (programmed cell death receptor-1) in peripheral blood mononuclear cells (PBMCs) isolated from normal weight (n=14), overweight (n=16) and obese (n=16) healthy blood donors. Percentage and MFI of TIGIT^{+} total NK cells (A), CD56^{bright}CD16^{dim} NK cells (B) and CD56^{dim}CD16^{bright} NK cells (C). Percentage and MFI of PD-1^{+} total NK cells (D), CD56^{bright}CD16^{dim} NK cells (E) and CD56^{dim}CD16^{bright} NK cells (F). Graphs are box and whisker plots with median ± minimum to maximum value; with additional dot plot representing individual donors. Overweight and obese groups were compared to normal weight control group.

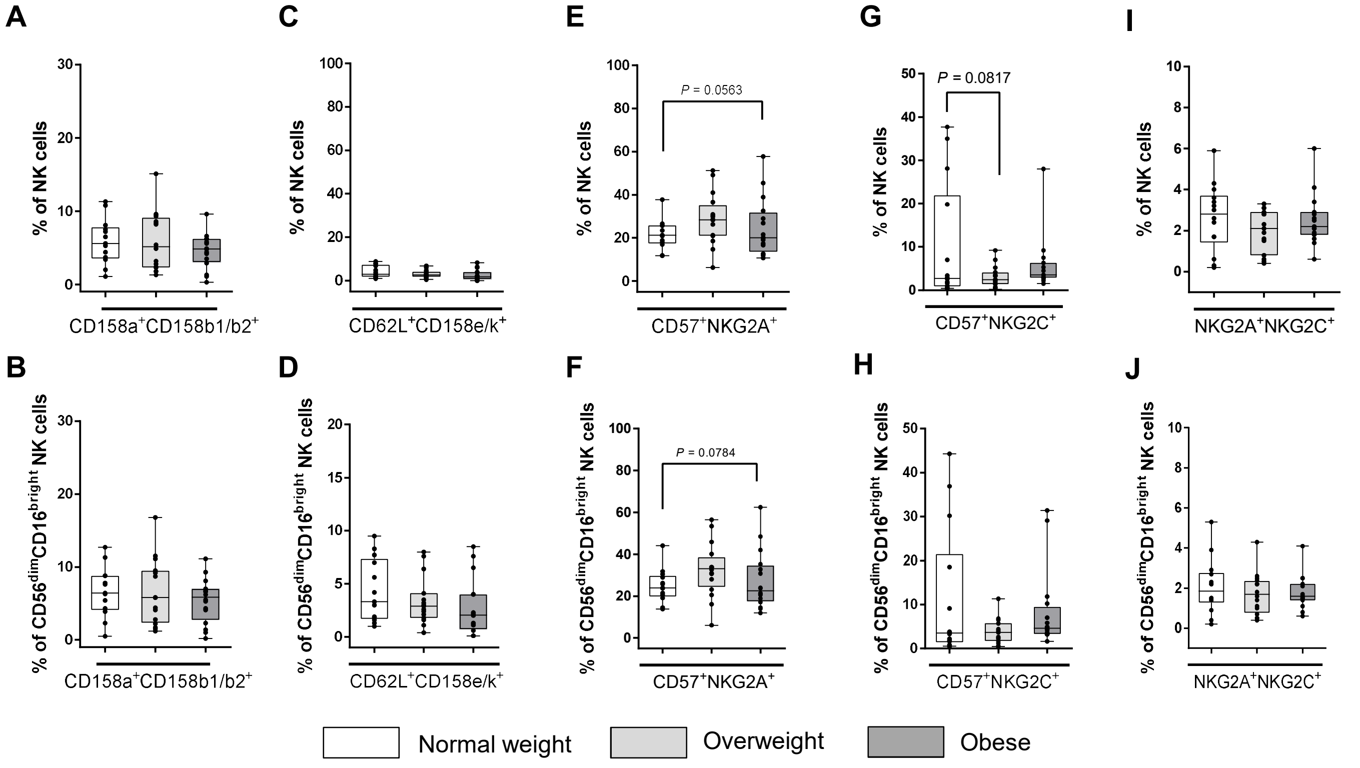

Supplementary Figure 6: Frequencies of total NK cells and $CD56^{dim}CD16^{bright}$ NK cells co-expressing inhibitory KIR receptors (A, B), adhesion molecule CD62L and inhibitory KIR receptor CD158e/k (C,D), maturation marker CD57 and inhibitory receptor NKG2A (E,F), CD57 and activating receptor NKG2C (G,H) and NKG2A and NKG2C (I,J) in peripheral blood mononuclear cells (PBMCs) isolated from normal weight (n=14), overweight (n=16) and obese (n=16) healthy blood donors. Graphs are box and whisker plots with median ± minimum to maximum value; with additional dot plot representing individual donors. Overweight and obese groups were compared to normal weight control group. P-values within $0.05 < P < 0.1$ are indicated with precise P-values.

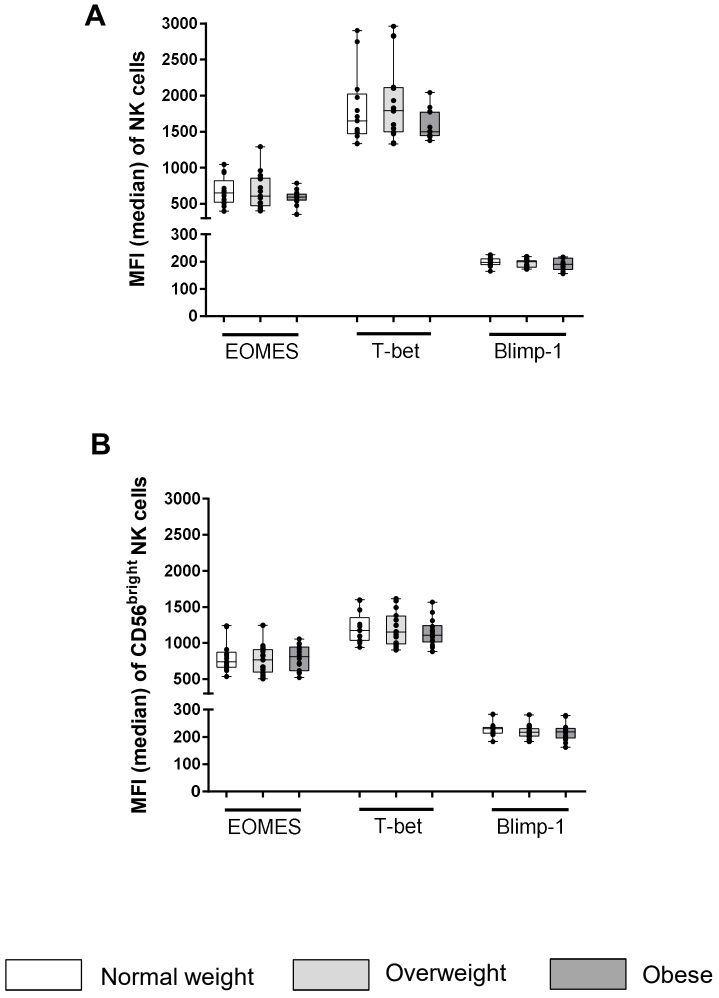

Supplementary Figure 7: Analyses of median fluorescence intensity (MFI) of the intracellular markers eomesodermin (EOMES), T-cell associated transcription factor (T-bet) and B lymphocyte-induced maturation protein-1 (Blimp-1) in total NK cells and CD56^{bright} NK cell subset of normal weight (n=13), overweight (n=15) and obese (n=13-14) individuals. Unstimulated PBMCs of blood donors with different body mass indexes (normal weight, overweight, obese) were stained with the respective fluorescent-conjugated antibodies. MFI of EOMES^{+}, T-bet^{+}, Blimp-1^{+} total NK cells (A) and CD56^{bright} NK cells (B). Graphs are box and whisker plots with median ± minimum to maximum value; with additional dot plot representing individual donors. Overweight and obese groups were compared to normal weight control group within each stimulation setting.

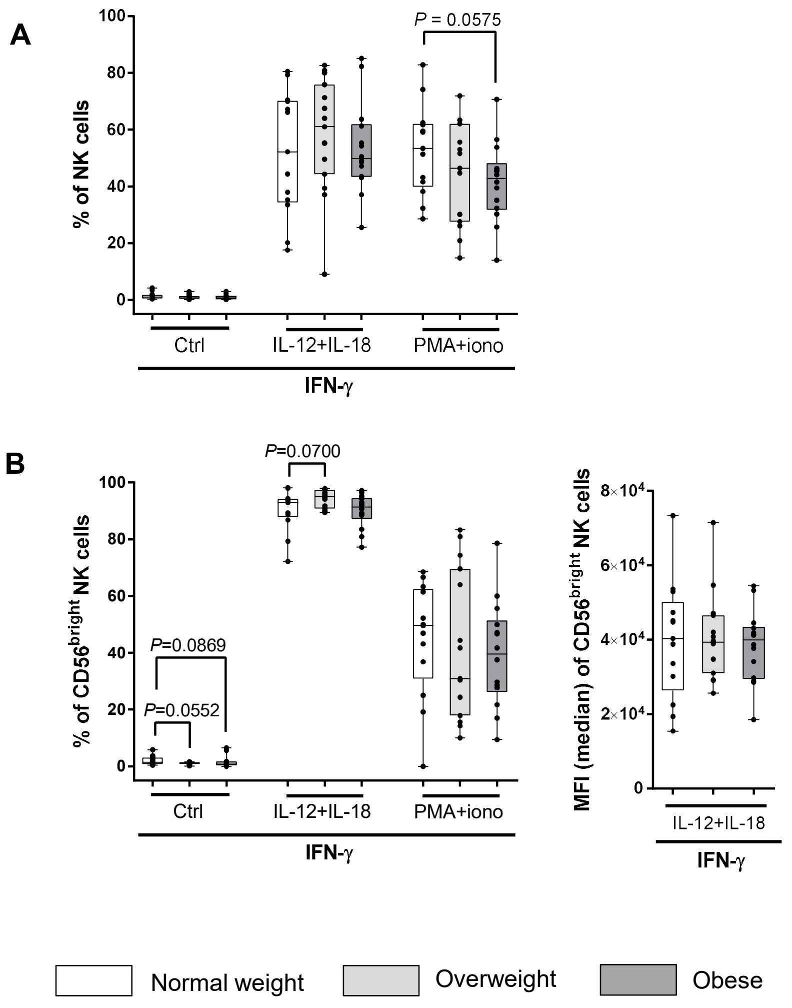

Supplementary Figure 8: Analyses of frequency of IFN-γ producing cells in total NK cells and CD56^{bright} NK cell subset as well as median fluorescence intensity (MFI) of IFN-γ^{+} CD56^{bright} NK cells of normal weight (n=13), overweight (n=15) and obese (n=14) individuals. PBMCs of blood donors were either stimulated with 10 ng/ml interleukin (IL)-12 in combination with 50 ng/ml IL-18 overnight or with 50 ng/ml phorbol-12-myristate-13-acetate (PMA) in combination with 1 µg/ml ionomycin for 4 h. Percentage of IFN-γ^{+} total NK cells (A) as well as percentage and MFI of IFN-γ^{+} CD56^{bright} NK cells (B). Graphs are box and whisker plots with median ± minimum to maximum value; with additional dot plot representing individual donors. Overweight and obese groups were compared to normal weight control group within each stimulation setting. P values within 0.05 < P < 0.1 are indicated with precise P-values. Ctrl: unstimulated control.

Supplementary Table 1: Fluorochrome-conjugated mononuclear antibodies for surface staining of human peripheral blood mononuclear cells for flow cytometric analysis.

**Antigen**	**Fluorochrome**	**Isotype**	**Clone**	**Concentration [µg/ml]**	**Company**
CD2	APC-H7	Mouse IgG1, κ	RPA-2.10	25.00	BD Biosciences
CD3	VioGreen	Mouse IgG2a, κ	BW264/56	44.00	Miltenyi Biotec
CD4	BV605	Mouse IgG1, κ	RPA-T4	50.00	BD Biosciences
CD8	PE	Mouse IgG1, κ	RPA-T8	6.25	BD Biosciences
CD14	FITC	Mouse IgG2b, κ	MϕP9	25.00	BD Biosciences
CD16	VioBlue	Human IgG1	REA423	55.00	Miltenyi Biotec
CD19	PE-Vio770	Mouse IgG1, κ	LT19	50.00	Miltenyi Biotec
CD25	PE-CF594	Mouse IgG1, κ	M-A251	50.00	BD Biosciences
CD27	PE	Hamster IgG, κ	LG.3A10	22.00	Miltenyi Biotec
CD56	APC	Human IgG1, κ	REA196	2.20	Miltenyi Biotec
CD57	BV 605	Mouse IgM, κ	NK-1	100.00	BD Biosciences
CD62L	PE-CF594	Mouse IgG1, κ	DREG-56	50.00	BD Biosciences
CD69	BV605	Mouse IgG1, κ	FN50	100.00	BioLegend
CD107a	PE	Mouse IgG1, κ	H4A3	3.00	BD Biosciences
CD158a (KIR2DL1)	PE-Vio770	Human IgG1, κ	REA284	88.00	Miltenyi Biotec
CD158b1/b2 (KIR2DL2/DL3)	PE	Mouse IgG2a, κ	DX27	27.50	Miltenyi Biotec
CD158e/k (KIR3DL1/DL2)	PE	Mouse IgG1, κ	5.133	77.00	Miltenyi Biotec
CD158i (KIR2DS4)	PE-Vio770	Mouse IgG1, κ	JJC11.6	88.00	Miltenyi Biotec
CD159a (NKG2A)	PE-Vio770	Human IgG1, κ	REA110	44.00	Miltenyi Biotec
CD159c (NKG2C)	PE	Human IgG1, κ	REA205	55.00	Miltenyi Biotec
CD161 (NK1.1)	BV605	Mouse IgG1, κ	HP-3G10	100.00	BioLegend
CD226 (DNAM-1)	BV605	Mouse IgG1, κ	DX11	200.00	BD Biosciences
CD244 (2B4)	PE-Cy7	Mouse IgG1, κ	C1.7	200.00	BioLegend
CD295 (OB-R)	PE-Vio770	Human IgG1, κ	REA361	8.25	Miltenyi Biotec
CD279 (PD-1)	BV605	Mouse IgG1, κ	EH12.2H7	100.00	BioLegend
CD314 (NKG2D)	PE-Vio615	Human IgG1, κ	REA797	100.00	Miltenyi Biotec
CD328 (Siglec-7)	PE	Mouse IgG1, κ	6-434	50.00	BioLegend
CD335 (NKp46)	PE-Vio615	Human IgG1, κ	REA808	16.50	Miltenyi Biotec
CD336 (NKp44)	PE-Vio770	Mouse IgG1, κ	2.29	16.50	Miltenyi Biotec
CD337 (NKp30)	PE-Vio615	Human IgG1, κ	REA823	5.00	Miltenyi Biotec
NKp80 (KLRF1)	PE-Vio615	Human IgG1, κ	REA845	37.50	Miltenyi Biotec
TIGIT (VSTM3)	BV605	Mouse IgG2a, κ	A15153G	200.00	BioLegend

APC, allophycocyanin; BV, brilliant violet; CD, cluster of differentiation; Cy, cyanine; DNAM, DNAX accessory molecule; FITC, fluorescein isothiocyanate; Ig, immunoglobulin; ITIM, Immunoreceptor tyrosine-based inhibitory motif; KIR, killer cell immunoglobulin-like receptor; Klr, killer cell lectin-like; NKG2, natural killer group 2; OB-R, obesity (leptin) receptor; PD-1, programmed cell death receptor-1; PE, phycoerythrin; Siglec, sialic acid binding immunoglobulin-like lectin; TIGIT, T-cell immunoreceptor with Ig and ITIM domains.
BD Biosciences, San Jose, CA, USA; BioLegend, San Diego, CA, USA; Miltenyi Biotec, Bergisch Gladbach, Germany.

Supplementary Table 2: Fluorochrome-conjugated monoclonal antibodies for intracellular staining of human peripheral blood mononuclear cells for flow cytometric analysis.

**Antigen**	**Fluorochrome**	**Isotype**	**Clone**	**Concentration [µg/ml]**	**Company**
TRAIL (CD253)	PE	Mouse IgG1κ	RIK-2.1	55.00	Miltenyi Biotec
IFN-γ	PE-Cy7	Mouse IgG1, κ	4S.B3	200.00	BD Biosciences
Granzyme B	Alexa Fluor 700	Mouse IgG1, κ	GB11	200.00	BD Biosciences
Granzyme A	FITC	Mouse IgG1, κ	CB9	25.00	BD Biosciences
EOMES	PE	Mouse IgG1, κ	WD1928	50.00	Thermo Fisher Scientific
T-bet	PE-Cy7	Mouse IgG1, κ	4B10	200.00	BioLegend
Perforin	PE-CF594	Mouse IgG2b, κ	δG9	50.00	BD Biosciences
Blimp-1	DyLight 488	Mouse IgG1	3H2-E8	600.00	Thermo Fisher Scientific

Blimp-1, B lymphocyte-induced maturation protein-1; CD, cluster of differentiation; Cy, cyanine; EOMES, eomesodermin; FITC, fluorescein isothiocyanate; IFN-γ, interferon-γ; Ig, immunoglobulin; PE, phycoerythrin; T-bet, T-cell associated transcription factor; TRAIL, tumor necrosis factor-related apoptosis-inducing ligand.
BD Biosciences, San Jose, CA, USA; BioLegend, San Diego, CA, USA; Invitrogen, Carlsbad, CA, USA; Miltenyi Biotec, Bergisch Gladbach, Germany; Thermo Fisher Scientific, Waltham, MA, USA.

Supplementary Table 3: Analyses of cytokine and chemokine secretion of peripheral blood mononuclear cells (PBMCs).

	Normal weight Mean ± SEM (n = 14)	Overweight Mean ± SEM (n = 16)	Obese Mean ± SEM (n = 16)
IFN-γ (pg/ml)	99.1 ± 35.6	51.9 ± 12.7	23.5 ± 2.8
TNF-α (ng/ml)	3.7 ± 1.3	1.5 ± 0.4	1.1 ± 0.2
GM-CSF (pg/ml)	267.4 ± 89.2	177.1 ± 52.2	107.8 ± 20.6
IL-6 (ng/ml)	7.8 ± 2.8	4.9 ± 1.5	3.3 ± 1.2
IL-10 (pg/ml)	255.6 ± 82.2	151.4 ± 35.3	104.0 ± 20.9
MIP-1α (ng/ml)	12.1 ± 2.8	11.1 ± 2.1	11.1 ± 2.0
MIP-1β (ng/ml)	14.5 ± 2.4	16.8 ± 1.7	16.0 ± 1.6
Granzyme A (pg/ml)	25.3 ± 2.9	25.6 ± 2.5	33.2 ± 4.0
Granzyme B (pg/ml)	724.3 ± 184.1	544.2 ± 99.1	483.1 ± 65.8
Perforin (pg/ml)	88.4 ± 13.2	94.0 ± 9.4	109.0 ± 11.1

GM-CSF, granulocyte-macrophage colony-stimulating factor; IFN-γ, interferon-γ, IL, interleukin; MIP; macrophage inflammatory protein; SEM, standard error of the mean; sFas, soluble Fas; sFasL, soluble Fas ligand; TNF-α, tumor necrosis factor-α.
